# Review of Participatory Epidemiology Practices in Animal Health (1980-2015) and Future Practice Directions

**DOI:** 10.1371/journal.pone.0169198

**Published:** 2017-01-17

**Authors:** Alberto Allepuz, Katinka de Balogh, Ryan Aguanno, Martin Heilmann, Daniel Beltran-Alcrudo

**Affiliations:** 1 Departament de Sanitat i Anatomia Animals, Facultat de Veterinària, UAB, Bellaterra, Barcelona, Spain; 2 UAB, Centre de Recerca en Sanitat Animal (CReSA, IRTA-UAB), Campus de la Universitat Autònoma de Barcelona, Bellaterra, Spain; 3 Animal Production and Health Division, Food and Agriculture Organization of the United Nations, Vialle delle Terme di Caracalla, Rome, Italy; Agencia de Salut Publica de Barcelona, SPAIN

## Abstract

In this study we combined an inventory of the major applications, geographic regions and diseases covered by participatory epidemiology (PE) activities in the field of animal health since 1980, together with an email discussion forum with PE practitioners from different regions of the world. The inventory included the search of peer-reviewed papers, master and technical reports, conference proceedings, manuals, training materials and projects. The search resulted in a low number of PE activity results until the year 2000, followed by a considerable increase (especially from 2012). Most of the identified activities were implemented in Africa and Asia, and focused on surveillance, disease survey and prioritization, and disease control. Seventy-nine PE practitioners working predominantly in Africa, Asia and Europe (29, 22 and 18 respectively) contributed to the email discussion forum. They proposed various modifications to the existing PE definition and discussed different issues related to the applicatoin of PE, its institutionalization for use in policy development, as well as the priorities for future development. The need to increase the number of PE trained people together with some methodological developments and the application of this methodology in developed countries, were some of the points highlighted during the forum. These factors stress the importance of further developing PE as a useful approach for engaging communities in addressing animal and related public health risks.

## Introduction

Participatory epidemiology aims to give a voice to communities while increasing our understanding of health problems and the options for their prevention, control and surveillance [1, 2]. The methodology originally emerged from medical anthropology and the participatory rural appraisal schools of thought, and thus far has mainly been applied in developing countries [3]. In the 1980s, participatory methods were used by veterinarians in community-based livestock projects in Africa and Asia [1]. Later, they continued to further develop and helped enhance the effectiveness of rinderpest surveillance in pastoralist systems in Africa [2]. There has been an increased interest in the participatory approach in recent years and it is considered an emerging field in veterinary epidemiology [3]. However, despite this growth, there is still not a standard definition for PE (http://www.participatoryepidemiology.info).

Though the central component of this methodology is the use of participatory techniques, the interpretation of the ‘participatory’ term requires differentiation between what is and what is not PE. For instance, Catley *et al*. [1] proposed that the term ‘participatory’ should include the active involvement of the community in the definition of objectives, interpretation of results and development of solutions. Also, in the context of surveillance, Toribio and Rushton [3] suggested that the participatory process involved in PE should empower stakeholders to solve their own problems and become actively involved in the surveillance system. In the last several years, numerous different projects and studies have used participatory methodologies. Catley *et al*. [1] provided a comprehensive review on the main strengths and weaknesses of PE in the field of animal health; however, there is not yet a systematic compilation of the activities performed. Moreover, some of the issues highlighted in that review may deserve further attention, such as the challenges due to possible conflicts of interest between community priorities and health policy rules, the institutionalisation of PE activities at the international and national level, priorities for further development, or the needed level of community involvement in order to discriminate what activities may be considered PE.

Therefore, the objectives of this study are to undertake an inventory of the major applications, geographic regions and diseases covered by PE activities in the field of animal health since 1980, and to discuss with PE practitioners different issues that deserve clarification.

## Materials and Methods

### Inventory of the major applications, geographic regions and diseases covered by PE activities in the field of animal health

A search of peer-reviewed papers, master or technical reports, conference proceedings, manuals, training materials, and past and present projects was performed as follows:

#### Search strategy

The language used for the search was English. The peer-reviewed papers to be included were those published from 1 January 1980 through 15 June 2015 (when the search was conducted). The literature search was carried out using the PubMed, Ovis and Web of Science bibliographic databases following the PRISMA guidelines for reporting systematic reviews [4] S1 File. The following set of key words (in English) was used: *(participatory AND (epidemiology OR surveillance OR outbreak OR control) AND animal)*. For the term animal, an exhaustive list of terminology (326 terms) referring to all major livestock and poultry species was used as described by Beltran-Alcrudo *et al*. [5]. On top of the most generic names, synonyms and scientific names, the list included additional terms based on expert opinion on the way livestock species of certain age, gender or neutered status are named. The identified livestock groups included bovines, camelids, equid, swine, poultry, rabbits and small ruminants.

After all records were sorted, each study was assessed for inclusion. The exclusion criteria were i) studies not focused on animal health issues (i.e. just human or plant health); ii) studies focused on animal health issues, but where non-participatory methods were employed; and iii) reviews with no original data. The selection process was conducted by screening the documents’ titles and abstracts. If eligibility remained ambiguous, the full text was then reviewed. Some of the papers not available (i.e. full text) through the bibliographic databases were downloaded through Google Scholar (http://scholar.google.com/).

In order to identify master, graduate theses, technical reports, conference proceedings, manuals and training material, the following websites were searched:

- Participatory epidemiology (http://www.participatoryepidemiology.info/).
- Participatory epidemiology network for animal and public health (PENAPH) (http://penaph.net/about/).

**Fig 1:**
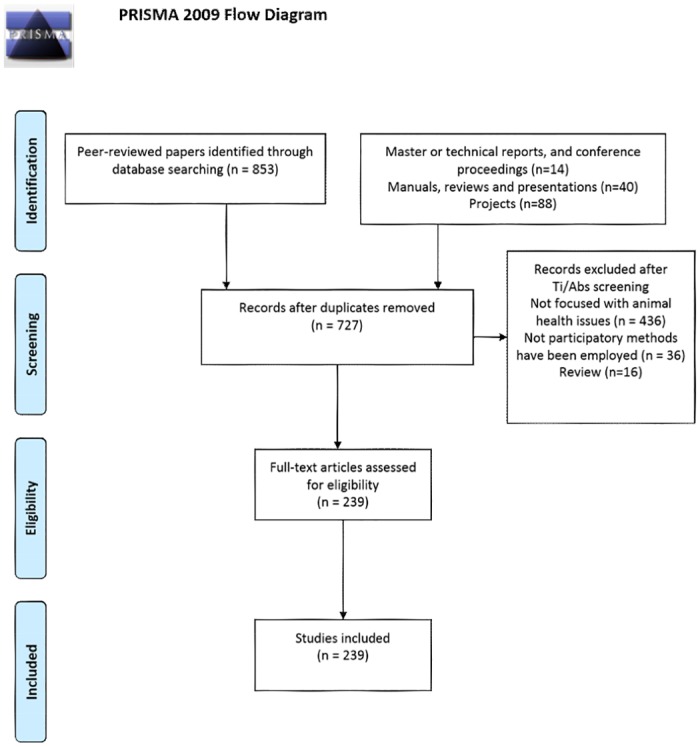
Flow diagram: search strategy steps.

Moreover, in order to identify past and present projects where PE was used as a methodology, focal points at selected institutions, universities, research groups and non-governmental organizations (NGOs) were contacted and requested the data described in Table 1. These people/institutions belonged to the authors’ networks of this manuscript, plus relevant authors identified through the paper selection process. This first set of electronic outreach (emails) led to additional contacts suggested by the contacted people, who were subsequently added to the list. This search was conducted until 10 December 2014. Fig 1 shows the steps applied in the search.

**Table 1:** Data fields extracted from the different items.

Type of item	Review question	Data
Peer-reviewed papers, master, graduate theses, technical reports and conference proceedings		
	General data	
		• Title of the study
		• Source: name journal or conference, master or technical report
		• Type: peer-review paper, report or conference proceeding
		• Year of publication
	What were the major applications of participatory epidemiology?	
		• Uses*
	What geographic regions and what diseases have been covered by participatory epidemiology?	
		• Disease(s)
		• Species
		• Country(es) where the study was conducted
Manuals, reviews and presentations		
	General data	
		• Title of the study
		• Year of publication
		• Type: manual, presentation, paper review or report
		• Reference
Projects		
	General data	
		• Project title
		• Year when the project started
		• Year when the project finished
	Which were the major applications of participatory epidemiology?	
		• Uses*
	What geographic regions and what diseases have been covered by participatory epidemiology?	
		• Disease(s)
		• Species
		• Country(es) where the study was conducted

*Uses: based on the veterinary uses of participatory epidemiology reviewed by [1] with some modifications.

#### Data extraction, management and analysis

From each selected item (i.e. peer-reviewed paper, conference proceeding, etc.) data was extracted in order to identify the major applications, geographic regions and diseases covered by PE activities. Table 1 shows a description of the data extracted from the different items.

Major applications were grouped based on the veterinary uses of PE reviewed by Catley *et al*. [1] with a few modifications. We added the applications ‘research’ (that was mainly applied for research projects) and ‘teaching’ (applied in the context of projects devoted to promote capacity building), and excluded ‘economics of veterinary service delivery’. The latter was merged with another category that included ‘economics’. Additionally, the ‘active surveillance’ application was replaced by ‘surveillance’, in order to use a more generic term that also included passive surveillance activities. Finally, we came up with a list of 11 applications: i) descriptive epidemiology; ii) disease investigation and diagnosis; iii) disease modelling; iv) disease survey and prioritization; v) economic or livelihood impact of disease; vi) evaluation of disease control methods; vii) evaluation of veterinary service delivery; viii) research; xix) surveillance; x) teaching; and xi) veterinary public health.

Regarding the diseases covered in PE activities, in those cases where the number of diseases was greater than two, general names were used, e.g. ‘food-borne diseases’, ‘vector-borne diseases’, or just ‘several diseases’ (when there was no common factor within which to group them). For studies that included multiple countries or whole regions, generic names were also used, e.g. ‘Horn of Africa’, ‘Sub-Saharan African countries’ or ‘South East Asia’. Three files were created and are available as supplementary material: Eligible peer-reviewed papers, graduate theses, master, technical reports, and conference proceedings (S2 File); Manuals, reviews and presentations (S3 File); and Projects (S4 File). An additional file with the excluded peer-reviewed papers, including the reason for their exclusion, was created (S5 File).

The descriptive analysis was conducted by using R software [6] and Quantum GIS [7]. Geographical data on the administrative boundaries for the whole world were obtained from a spatial database named GADM (http://www.gadm.org/).

### Expert discussion

This study did not need any ethical approval as it did not included samples or experiments on people. It only included the expression of opinions in relation to a specific topic and the decision to participate or not was solely from each person. Moreover, all data was anonymously analysed.

Following the criteria for reporting qualitative research (Tong *et al*. [8] and S6 File) the discussion with PE practitioners followed this procedure:

#### Research team and flexibility

The authors of this paper moderated the discussions. No relationship between the authors of this paper and the participants was established prior to the discussion, despite some of them might know the moderators due to their participation in other forums or projects.

#### Study design

An email list server (PE@FAO.org) was launched from FAO and an introductory email explaining the structure of the email discussion and the weekly topics to be discussed was sent. Recipients included all the people involved in PE activities identified during the previous inventory, and attendance lists to PE workshops and training courses as provided by some collaborators. Moreover, in order to ensure that additional interested experts had the opportunity to join, the email discussion forum was announced through 1) PENAPH website (http://penaph.net/), which is a network aimed to connect groups and individuals who apply PE methods; 2) FAO’s Veterinary Public Health e-bulletin; and 3) the Epivet mailing list which is a discussion forum for veterinary epidemiologists.

A total of 79 people contributed to the email discussion and data was collected at workplace. In terms of the region of the participants, 37% (i.e. 29 out of 79) worked in Africa; 18% (14 out of 79) in Asia; 16% (13 out of 79) in Europe; 10% (8 out of 79) in America; and one person in Oceania. The other 14 people did not provide details about their country or regional scope. In relation to the type of institution that they were involved with, 47% (37 out of 79) worked at a university or a research centre; 16% (13 out of 79) in the official veterinary services of the government of their country; 14% (11 out of 79) worked for FAO; three where independent consultants; and one of the contributors worked for an NGO. The other 14 did not provide information on the type of institution where they worked. No reasons were provided by the participants about their non-participation decision neither why some of them dropped out during the email discussions.

The themes to be covered were provided by the authors to the forum and the email discussion was held during a period of three weeks, lasting from 12 to 30 of October 2015, each week discussing a new theme. The weekly topic was presented on Monday, and on Wednesday a summary of responses received was released to the forum and additional questions or comments in order to encourage further debate. On Friday, the weekly discussion was closed and comments were compiled.

#### Analysis and findings

We did not performed data coding neither the use of any software package for analysis. We summarized the responses received by mid-week so participants had opportunity to feedback on the findings. Themes to be discussed were first defined by the authors of this paper based on their previous experiences during the inventory of PE activities and then further discussed with several of the PE experts identified during the inventory.

The details about the topics of the weekly discussions were as follows:

- Topic 1: Discussion about PE definitions and examples of its successful application.

We provided two definitions and participants were asked to select the preferred definition, attempt to improve it, or to provide an alternative definition.

I *“Participatory epidemiology is an emerging field that is based on the use of participatory techniques for the harvesting of qualitative epidemiological intelligence contained within community observations*, *existing veterinary knowledge and traditional oral history”* [9].
II *“Participatory epidemiology is the systematic use of participatory approaches and methods to improve understanding of diseases and options for animal disease control*. *The term ‘participatory’ should be used to refer to the active involvement of communities in the definition of project objectives and development of disease control strategies and therefore should go beyond the simple provision of information to outsiders”* [1].

In addition, participants were asked to provide examples of PE with which they were familiar. Finally, three examples were provided and participants were asked to express their views on whether or not they considered them an example of a PE activity. The three examples were:

I *Social factors influencing the eradication of bovine tuberculosis (bTB) in Spain*: This project used semi-structured interviews with key people, representative of the different sectors involved in the bTB program (i.e. private and official veterinarians and farmers). Those issues identified in the exploratory interviews were further investigated through qualitative in-depth interviews from a sample of different types of stakeholders. The importance of the different social factors was quantified through a representative quantitative survey.
II *Knowledge, attitudes, and practices concerning Middle East respiratory syndrome (MERS) among Umrah and Hajj pilgrims in Samsun, Turkey, 2015 [10]:* They performed a questionnaire to determine knowledge, attitudes, and practices concerning Middle East respiratory syndrome (MERS) among people intending to participate in the Hajj or Umrah pilgrimages.
III *Risk of introduction of Rift Valley fever and foot and mouth disease in Egypt through animal movements*: to estimate the number of animals that enter Egypt, both legally and illegally, and describe the animal movement patterns, informal interviews with key persons who potentially have knowledge on illegal trade was performed. The work was performed in coordination with the veterinary services of Egypt.

- Topic 2: How to institutionalise PE activities to allow for their use in policy development at various levels (e.g. national and international).

At the second week, participants were asked about two issues:

I How to incorporate PE activities within official surveillance systems, e.g. how to allow for laboratory diagnosis of PE findings, or how to combine with other (passive or active surveillance) approaches?
II How to deal with possible conflicts of interest between community priorities and animal health policy, e.g. diseases important for the community that are not prioritized by the vet services?

- Topic 3: What are the priorities for future PE developments?

During this week, no specific question was launched and it was left in an open format so that the scope of the answers would not be restricted.

All the original comments have been included as supplemental material (S7 File) to ensure readers have the opportunity to assess them without possible interpretation bias from the authors. Names have been deleted from the contributions in order to ensure anonymity, but the names of all those that have contributed are included in the ‘acknowledgments’ section.

## Results

### Inventory of PE activities

**Fig 2:**
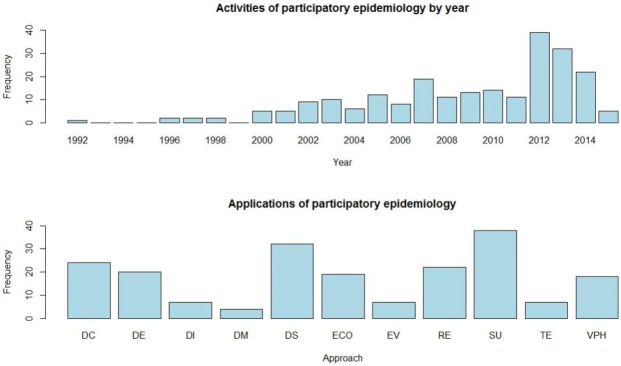
Number of participatory epidemiology activities by year and applications. DC: Evaluation of disease control methods; DE: Descriptive epidemiology; DI: Disease investigation and diagnosis; DM: Disease modelling; DS: Disease survey and prioritization; ECO: Economic or livelihood impact of disease; EV: Evaluation of veterinary service delivery; RE: Research; SU: Surveillance; TE: Training; VPH: Veterinary Public Health.

A total of 237 PE activities were identified through this review: 110 peer-reviewed papers, master, graduate thesis, technical reports or conference proceedings; 39 manuals or training materials and 88 projects (S2, S3 and S4 Files). Fig 2 presents the number of PE activities by year and their applications. Until the year 2000, the number of reported PE activities was limited, but increased considerably thereafter, especially from 2012. The more common uses of PE activities (excluding manuals and training material) were for ‘Surveillance’ applications (20%, 39 out of 198), followed by ‘Disease survey and prioritization’ and ‘Evaluation of disease control methods’ (16% and 12%, respectively).

**Fig 3:**
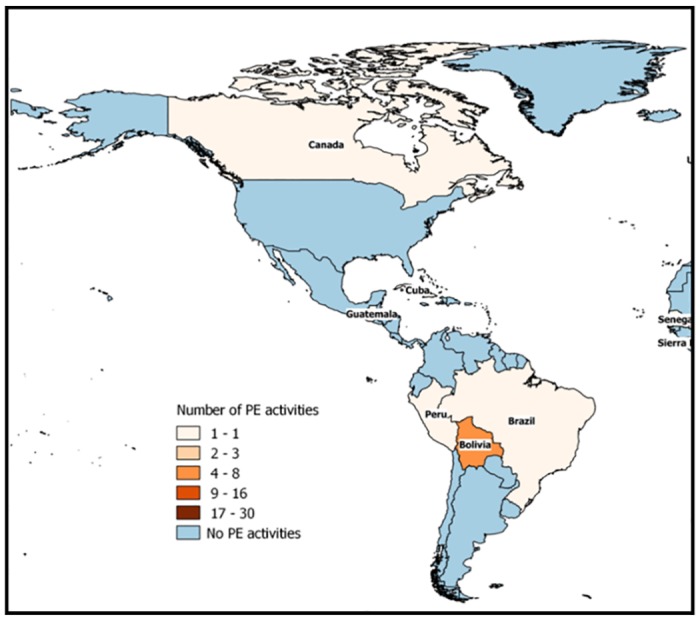
Number of participatory epidemiology activities in America.

**Fig 4:**
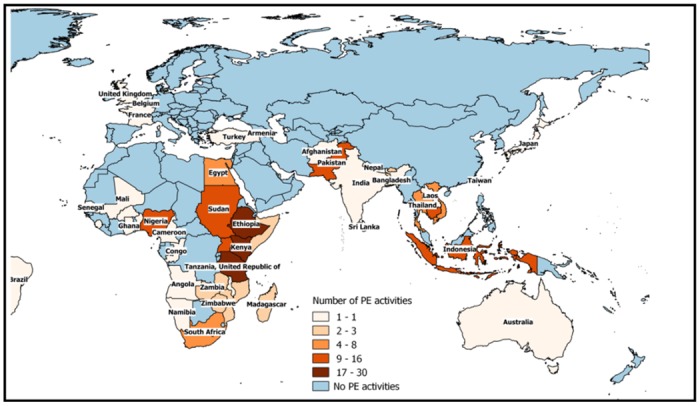
Number of participatory epidemiology activities in Europe, Africa, Asia and Oceania.

PE activities were conducted in 52 different countries, most of them in Africa (48%, 25 out of 52), followed by Asia with 33% (17 out of 52). In America, Europe and Oceania, just six, three, and one PE activities where identified through this review (Figs 3 and 4).

Table 2 describes the diseases covered by the peer-reviewed papers, graduate thesis, master, technical reports or conference proceedings and by the projects identified through this inventory. It has to be taken into account that in those cases that two diseases were covered by the PE activity both of them were taken into account and when the number of diseases was greater than two general names were used. Because of that, the total number of diseases covered is greater than the number of papers or projects identified during the inventory. Most of this studies targeted multiple diseases (57 out of 207), followed by avian influenza (22 out of 207) and foot and mouth disease (19 out of 207).

**Table 2:** Diseases covered within the participatory epidemiology activities.

Diseases	Papers*	Projects	Total
Several**	37	20	57
Avian influenza (AI)	12	10	22
Foot and mouth disease (FMD)	12	7	19
*Food borne diseases*	6	8	14
Trypanosomiasis	11		11
Rinderpest	5	2	7
Newcastle disease (ND)	3	3	6
Rabies	2	4	6
Contagious bovine pleuropneumonia (CBPP)	4	1	5
Rift Valley fever (RVF)	1	4	5
Cryptosporidiosis	4		4
Brucellosis		3	3
Intestinal helminth infections	2	1	3
Peste des petits ruminants (PPR)	2	1	3
Tick-borne diseases	1	1	2
African swine fever (ASF)		2	2
Anthrax	1	1	2
Contagious caprine pleuropneumonia (CCPP)	1	1	2
*Zoonoses*		2	2
Anaplasmosis	1		1
Babesiosis	1		1
Bovine dermatophilosis	1		1
Campylobacter infection		1	1
Classical swine fever		1	1
Crimean-Congo haemorragic fever (CCHF)		1	1
East Coast fever (ECF)		1	1
Infectious bovine keratoconjunctivitis		1	1
Lameness		1	1
Liver fluke	1		1
Mastitis	1		1
Neosporosis	1		1
Q fever	1		1
Scabies	1		1
Schistosomiasis	1		1
Schmallenberg		1	1
None	2	14	16
*Total*	115	92	207

*Includes peer-reviewed papers, graduate thesis, master, technical reports and conference proceedings.

**Several: more than two diseases, which could not be categorized with a generic name such as food-borne, etc. *Italics* for groups of diseases.

### Expert discussion

#### Discussion about a PE definition

Fifty-eight comments were received related to this issue (S7 File). In general, preference was given to the definition provided by Catley *et al*. [1], as participants highlighted that it conveys a clearer statement about the importance of the participatory aspect of PE. However, several modifications were proposed:

- To include expressions that clarified the participatory nature of this methodology, such as ‘the empowerment of people to identify and solve their own problems’ or ‘the shared learning environment generated through the application of participatory techniques’.
- Several suggested to delete the term ‘participatory’ from the definition in order to avoid circular arguments, while others suggested to replace it with the term ‘active involvement’. However, some contributors expressed concerns about this as they found it important to clarify the term ‘participatory’ as part of the definition since it is a term that is often misunderstood. Based on these comments we decided not to include the term ‘active involvement’ within the definition of ‘participatory’, but rather to stress in the definition that PE should promote the participation of people.
- To include the words ‘population’ (as epidemiology is the study of health and disease in populations) and ‘evaluation’ (as PE is also used to evaluate disease control) and to delete the word ‘project’ (as it is not necessary to originate from a project).
- To replace ‘animal disease control’ with the word ‘health’ in order not to limit the definition to animal health, as PE is also used to assess health risks linked with pollution, farm management, waste management, wildlife populations and loss of biodiversity, among others.
- The use of the word ‘communities’ raised several comments. Some people suggested replacing it with the expression ‘all stakeholders involved’ while others pointed out that these two terms are not really synonymous and that PE could target groups of people which would not fit the definition of a community. Therefore, we decided not to restrict PE’s definition to a community or to all the stakeholders and to instead insert a generic term such as ‘people’.
- To include points not covered in the definition, such as the idea that PE is founded on the principle of equal partnership with mutual respect and trust, that it should ensure acceptability and sense of ownership, and to make clear that PE should help to create a better understanding of people’s perceptions about risk or health problems.

Based on all of the above suggestions, we came up with the following Catley *et al*. [1] modified definition:

“*Participatory epidemiology is the systematic use of approaches and methods that facilitate the empowerment of people to identify and solve their health needs. It should promote the participation of people leading to a shared learning environment that improves the understanding of their risk perception, health risks and options for surveillance, control, and health evaluation in populations. It should be conducted by professionals on equal partnership among all involved in the activity and with mutual respect and trust, ensuring acceptability and a sense of ownership*”.

#### Examples of PE applications

Seventeen participants provided examples of applications related to areas, including disease investigation, control, surveillance, and descriptive epidemiology. However, there were not many comments highlighting the strong and weak points of this methodology. Most examples belonged to applications in Africa and South-East Asia, but interestingly, a couple of cases referred to Europe. Participants highlighted the valuable contribution of integrating community observations in disease investigation and control activities in order to increase the success of these activities. It was also pointed out that PE has more ability to capture emerging and re-emerging diseases compared to traditional techniques, and enables the community to provide and receive immediate feedback on recommended precautionary/prevention measures. The role of PE in appreciating the limitations of disease spread models and thus being a valuable tool to validate them and understand the disease pattern by the researcher and the community (i.e. veterinarians and farmers in this example) was highlighted. The ability of these tools to look at complex eco-health issues and solutions was also mentioned. Finally, some examples in Europe provided from a surveillance standpoint identified increased trust and acceptability of the system by ensuring that all stakeholders had a voice in the development of such programs.

Areas to be improved included the fact that PE is a flexible tool, and therefore there is a risk of creating data by sitting in the office without visiting communities, as well as that budget and time constrains could limit the involvement of all the stakeholders. The lack of statistical analytical tools useful to present the enormous quantity of data generated during PE activities was also pointed out.

#### Discussion about the provided examples in topic 1 of the discussion

Sixteen participants expressed their views in relation to whether we should consider the provided examples as a PE activity. The first and third examples were considered as a PE activity 14 times each and the second example 4 times. Reasons for considering an example as a PE activity were mostly due to the use of participatory techniques and the involvement of different stakeholders, while the use of a traditional questionnaire was the main reason not to consider the example as a PE activity.

#### How PE activities could be institutionalized to allow for their use in policy development

We received 41 comments for this issue. Several of them highlighted the need to increase the number of trained people through the incorporation of this methodology in undergraduate programs, and the organization of different training activities such as courses for the public veterinary services or field veterinarians. It was also advised to promote a greater involvement of the research community, as this would facilitate the dissemination of PE knowledge into institutions, thereby increasing available training opportunities. In addition, a more active involvement of the research community was perceived as a way to provide inputs on the limits and strengths of this methodology and was viewed as a way to promote its use. Another route discussed to promote its use was by disseminating (through relevant case studies) the merits of PE among different sectors, such as veterinary services, policy makers, international organizations or farmers. Laboratory diagnosis or further assessment/investigation were also identified as ways to address the need to validate results from PE activities. Also enhancing communication between farmers and those responsible for surveillance by using new mobile technologies could facilitate the use of PE within the surveillance programs.

The lack of national institutional frameworks and international standards for the implementation and for the implementation and validation of PE activities was considered an important limiting factor in achieving PE’s institutionalization. In this sense, it was suggested that the World Organisation for Animal Health (OIE) and the Food and Agriculture Organization of the United Nations (FAO) should develop international standards, guidelines and manuals for the veterinary authorities. This should facilitate the integration of PE with other existing surveillance techniques and ensure that it is guided by professionals.

Finally, different examples within which PE activities had been successfully institutionalized were provided by the participants. They included the case of Rinderpest surveillance in Pakistan and Kenya, and the avian influenza control and prevention program in Nigeria. On the other hand, some weaknesses were also mentioned, for example in Egypt, where participatory disease surveillance (PDS) showed an increase in the sensitivity for the detection of highly pathogenic avian influenza (HPAI) cases, but also resulted in a decrease in the specificity. Also, factors such as insufficient numbers of trained practitioners and a lack of both support and funding to continue PE activities limited the incorporation of PE into official programs.

#### How to deal with possible conflicts of interest between community priorities and animal health policy, e.g. diseases important for the community that are not prioritized by the veterinary services

Most of the 49 comments received attributed the existence of conflicts of interests to a lack of a proper understanding of the community needs. The reflection of community priorities was seen by several contributors as something mandatory, since these policies should be stakeholder-based in order to ensure the support and sustainability of the system. In this sense, PE methodology was seen as a tool that could fill this gap, as it should enable a closer contact with farmers and an opportunity to organise their concerns. The application of PE techniques in the development of animal health policies was therefore encouraged to incorporate community interests in such policies and achieve successful programs.

Nevertheless, it was also mentioned that some conflicts may be unavoidable, such as 1) cases of disease that may not have a large direct impact in animals (and thus may not be a priority for farmers), but still have public health effects (e.g. H7N9 avian influenza or Hepatitis E); 2) the fact that communities perceive only immediate losses, while animal health policy has both a wider and longer term view (e.g. diseases with trade implications) or 3) regional differences in community priorities across a country, as animal health policies are normally set at the national level.

The organization of workshops or other communication activities in order to clearly explain animal health policies and therefore educate stakeholders was seen as an important point to reduce conflicts. Addressing high-priority health problems of the community together with the implementation of animal health policy was mentioned as a possible way to manage these conflicts. This was illustrated by the example of the highly pathogenic avian influenza (HPAI) control program in Africa, which, to be effective, had to incorporate all poultry diseases that are differential diagnoses for HPAI. Finally, the importance of establishing decentralized animal health policies in order to accommodate regional differences was also mentioned. Additional thoughts focused on the need for more training and capacity building to build a bigger PE workforce. The promotion of the use of PE methods in developed countries was perceived as an important way to draw attention to PE and encourage the use of such approaches in other countries. Some weaknesses were mentioned related with 1) the possible conflict of community animal health workers to report a disease in their village that could have negative consequences for their community; 2) the difficulty to analyze the data generated in PE activities, which could lead to loss of interest in using the method (and therefore, the need to train users in managing and analyzing data); 3) the possible conflict between the interest of the international community and animal health policy within a country; and 4) financial and practicability constraints to implement actions derived from PE exercises.

#### What are the priorities for future PE developments?

For this last topic, we received 8 comments indicating the need for more training courses, manuals, and the incorporation of PE in the curricula of veterinary schools. Other priorities were related to methodological issues, such as the need to validate PE methods by comparing them to conventional epidemiological practises, and the development of stronger analytical tools adapted to PE. Other contributors suggested to develop a checklist that could guide the application of PE and to provide information on feasibility, accuracy and costs/benefits. Finally, some political suggestions were provided to enhance the integration of PE in official veterinary services, including lobbying in FAO and OIE, and for these two institutions to promote PE among national veterinary services.

## Discussion

In performing this work, we collected data about PE activities that have been conducted in the field of animal health, together with the opinion of PE practitioners concerning different issues. Though PE methods are being used in other fields such as public or environment health, it was decided to concentrate on addressing the field of animal health in order to focus the literature search and try to discuss the particularities of PE within this field. In the future, it would be desirable to expand this work to further studies in the public and environment health fields in order to get the full picture of activities performed within PE.

Results from this work have to be interpreted taking into account the likely existence of different selection biases due to the procedure used in the literature search, and different factors that might have influenced the participation in the email discussion forum. The more evident one is related to the language used: the literature search was performed using the English language and by doing so we could only choose materials published in that language. Further extensions of this study could include a literature search using other languages. Moreover, part of the literature search was based on peer-reviewed papers and therefore all those studies using PE as a methodology which have not been published or were not included in a technical report, manual, or conference proceeding would not have been identified. In addition, the procedure used to identify past and present projects where PE was used as a methodology was mainly based on replies of the people contacted by email. It could be the case that some did not answer due to time constraints or that relevant practitioners involved in PE projects were not identified. We tried to contact as many individuals as possible; however, we acknowledge that some projects will not have been captured in this review. In the email discussion we had contributions from PE practitioners from different parts of the world, however it has to be taken into account that several PE practitioners might not feel comfortable discussing in English and, therefore, the outcome of the email discussion was clearly biased. In order to simplify the implementation of the discussion, we decided to assume this selection bias; however, it would be a useful exercise to conduct further discussions about the issues raised in this paper at the local level and in the local languages to compile a more complete picture.

One of the most debated issues was the definition of PE, from which we received a total of 58 comments. Several modifications were offered by participants to the definition proposed by Catley *et al*. [1] and we tried to take all of them into account. Based on the comments received, we believe we were able to incorporate some useful adjustments. In addition to the suggested modifications, we also received some alternative PE definitions and modifications to the older definition proposed by Mariner *et al*. [9]. In order to simplify the analysis we decided to focus on the proposal that had the greatest support, as we did not feel that it was possible to compile all the different comments into a single definition. Nevertheless, we acknowledge the difficulties in such a task and we have provided the original comments in their entirety in the S7 File to enable the readers to review them.

In relation to the results from the inventory of PE activities, it has to be taken into account that the included activities may not always fit with the definition proposed above. The identified material was not always clear enough to allow for the assessment of the real involvement of the community, and therefore some of the activities identified might not be considered to be truly PE activities. This was also mentioned in the review by Catley *et al*. [1], where there were doubts about whether some of the included studies would match the proposed ‘participatory approach’. The opposite may also happen, when papers failed to clearly describe the PE approaches used and, hence, failed to be incorporated into this study. What is and what is not PE seems to be a critical aspect, and despite the previously reported working definitions, as evidenced by the lack of consensus to classify the three provided examples, the critical question of what is and what is not PE might not be clear enough. It is acknowledged that there was not enough data on these examples in order to assess if they could be considered as PE, but despite this fact, the second example that was just an assessment of knowledge, attitudes and practices by using a structured questionnaire, was considered as a PE activity by several based on the assessment of social factors. In this sense, it might be useful to differentiate between social epidemiology, which aims to study the impact of social factors on health, and PE, which seeks a more active participation of people.

The inventory of activities showed a clear increase in PE since 2000, which matches with the classification of PE as an emergent field [9]. The decrease observed in 2014 is, in the authors’ opinion, artificial and most likely related to the date of a peer-review acceptance or concession of a project being delayed in relation to the date when the activity was planned or performed. Moreover, for 2015 we only searched until 15 June 2015, so the results of this year are not comparable with previous years’ results.

The need of more trained professionals in PE was repeatedly mentioned during the email forum discussions, and the inclusion of PE in the veterinary university curricula has been suggested as a possible remedy. This reflection was already elaborated by Toribio and Rushton [3] when deciding whether or not to include PE in the membership examination in epidemiology, asking themselves whether PE could be considered an accepted component of epidemiology practice. As described in that paper, PE is considered a young discipline and some aspects such as a critical analysis of the strengths and weaknesses, or examples of how participatory methods could be combined with conventional tools to add value to decision making processes might deserve deeper revision. An attempt was made to cover some of these issues during the email discussion. However, despite some weaknesses being mentioned, there was not a deep debate about them. In our opinion, this was influenced by most participants being very keen in promoting the use of PE and therefore placed more emphasis on the positive aspects rather than the negative ones. A greater involvement of the research community in PE activities was suggested during the email discussion to provide inputs on the limits and strengths of this methodology. It was remarkable that a high percentage of the contributors to this discussion worked at research centres or universities, which could suggest that this is currently being developed and new advances within this methodology might be expected in the near future. Contributors also suggested that OIE and FAO should develop international standards, guidelines and manuals for the veterinary authorities in order to apply PE. In this sense, it is worthwhile mentioning that a recent surveillance manual launched by the OIE [11] has included a chapter where PE is described and that there are already quite a large number of manuals and training materials available on the web (S3 File).

The discussion about the application of PE in developed countries was also an interesting topic which arose from the email discussion forum. As mentioned by some participants, PE is often assumed by many to be applicable only for developing countries. However, this ideology is unfounded, and in our opinion, PE could be equally useful in developed countries. Indeed, participants provided some examples about PE applications in developed countries, related with the improvement of surveillance in small ruminants or in the process of developing animal health policies. However, these examples were the exception and most referred to Africa, Asia, and Central and South America. Further explorations of PE applications in developed countries might be desirable.

PE has been reported to produce limited numerical data limiting its credibility for quantitative epidemiologists [3]. During the email discussion forum, the need for statistical methodological developments to use for PE results was mentioned several times. In this sense, a limitation could be that more complex analyses would make it difficult to share results with the community, leading to PE becoming a more conventional research tool rather than a participatory approach [1]. In spite of this, there seems to be a methodological avenue of research within the PE field.

Finally, and despite the different limitations and selection bias inherent to this review, we hope to have been able to give a useful view on the PE activities that have been performed, together with interesting opinions about different issues related to PE in the field of animal health.

## Conclusions

The inventory of PE activities conducted during this study revealed a low number of activities until the year 2000 with a considerable increase since, especially from 2012. Most of the identified activities were implemented in Africa and Asia, and focused on surveillance, disease survey and prioritization, and disease control. Based on the suggestions provided during the email discussion forum, we proposed some modifications to the working PE definition previously reported by Catley *et al*. [1]. The need to increase the number of PE trained people, together with some methodological developments, exploring avenues to institutionalize PE, and discussing the application in developed countries were points highlighted during the forum and point to the importance of further developing PE as a useful approach for engaging communities in addressing animal and related public health risks.

## Supporting Information

S1 File PRISMA guidelines for reporting systematic reviews. (PDF)

S2 File Eligible peer-reviewed papers, graduate theses, master, technical reports, and conference proceedings. (XLS)

S3 File Manuals, reviews and presentations. (XLS)

S4 File Projects. (XLS)

S5 File Excluded peer-reviewed papers, including the reason for their exclusion. (XLS)

S6 File Criteria for reporting qualitative research. (PDF)

S7 File Original comments from the email discussion forum. (PDF)
