# Supplementary material for: Review of Participatory Epidemiology Practices in Animal Health (1980-2015) and Future Practice Directions
Source: PLoS One. 2017 Jan 17;12(1):e0169198. doi: 10.1371/journal.pone.0169198 (PMC5240953; doi:10.1371/journal.pone.0169198)
Supplement: S6 File — (PDF) [file pone.0169198.s006.pdf]

Item	Guide questions/description	Reported on page #
**Domain 1: Research team and reflexivity**		
**Personal Characteristics**		
1. Interviewer/facilitator	Which author/s conducted the interview or focus group?	5
2. Credentials	What were the researcher’s credentials? E.g. PhD, MD	5
3. Occupation	What was their occupation at the time of the study?	5
4. Gender	Was the researcher male or female?	5
5. Experience and training	What experience or training did the researcher have?	5
**Relationship with participants**		
6. Relationship established	Was a relationship established prior to study commencement?	5
7. Participant knowledge of the interviewer	What did the participants know about the researcher? e.g. personal goals, reasons for doing the research	5
8. Interviewer characteristics	What characteristics were reported about the interviewer/facilitator? e.g. Bias, assumptions, reasons and interests in the research topic	5
**Domain 2: study design**		
**Theoretical framework**		
9. Methodological orientation and Theory	What methodological orientation was stated to underpin the study? e.g. grounded theory, discourse analysis, ethnography, phenomenology, content analysis	NA
**Participant selection**		
10. Sampling	How were participants selected? e.g. purposive, convenience, consecutive, snowball	5
11. Method of approach	How were participants approached? e.g. face-to-face, telephone, mail, email	5
12. Sample size	How many participants were in the study?	6
13. Non-participation	How many people refused to participate or dropped out? Reasons?	6
**Setting**		
14. Setting of data collection	Where was the data collected? e.g. home, clinic, workplace	6
15. Presence of non-participants	Was anyone else present besides the participants and researchers?	NA
16. Description of sample	What are the important characteristics of the sample? e.g. demographic data, date	6

**Data collection**		
17. Interview guide	Were questions, prompts, guides provided by the authors? Was it pilot tested?	6
18. Repeat interviews	Were repeat interviews carried out? If yes, how many?	NA
19. Audio/visual recording	Did the research use audio or visual recording to collect the data?	NA
20. Field notes	Were field notes made during and/or after the interview or focus group?	NA
21. Duration	What was the duration of the interviews or focus group?	6
22. Data saturation	Was data saturation discussed?	NA
23. Transcripts returned	Were transcripts returned to participants for comment and/or correction?	6
**Domain 3: analysis and findingsz**		
**Data analysis**		
24. Number of data coders	How many data coders coded the data?	6
25. Description of the coding tree	Did authors provide a description of the coding tree?	6
26. Derivation of themes	Were themes identified in advance or derived from the data?	6
27. Software	What software, if applicable, was used to manage the data?	6
28. Participant checking	Did participants provide feedback on the findings?	6
**Reporting**		
29. Quotations presented	Were participant quotations presented to illustrate the themes / findings? Was each quotation identified? e.g. participant number	7
30. Data and findings consistent	Was there consistency between the data presented and the findings?	7
31. Clarity of major themes	Were major themes clearly presented in the findings?	7
32. Clarity of minor themes	Is there a description of diverse cases or discussion of minor themes?	7

From: Tong A, Sainsbury P, Craig J. Consolidated criteria for reporting qualitative research (COREQ): a 32-item checklist for interviews and focus groups. Int J Qual Health Care. 2007; 349-357.
