# Supplementary material for: Review of Participatory Epidemiology Practices in Animal Health (1980-2015) and Future Practice Directions
Source: PLoS One. 2017 Jan 17;12(1):e0169198. doi: 10.1371/journal.pone.0169198 (PMC5240953; doi:10.1371/journal.pone.0169198)
Supplement: S7 File — (PDF) [file pone.0169198.s007.pdf]

Topic 1

Comments related to the definition of PE

1.

This is a perfect definition, "Participatory epidemiology is an emerging field that is based on the use of participatory techniques for the harvesting of qualitative epidemiological intelligence contained within community observations, existing veterinary knowledge and traditional oral history."

2.

The two definitions are concise and good enough but may be made better if combined and some other key words (like emerging) re-evaluated. I believe strongly that PE existed long before being recently considered. I also feel that PE definition may be incomplete without mentioning the key players and the advantage of the approach, I thus suggest "Participatory epidemiology (PE) is a re-emerging branch of veterinary epidemiology which is based on participatory rural appraisal methods that involves key stakeholders to improve both the understanding as well as developing the best and sustainable options for livestock disease control".

3.

I agree with these two definitions and especially the second (that of Catley et al.). Participatory epidemiology is an approach to better know the diseases in order toeffectively control them. The involvement of all stakeholders (participatory aspect) is fundamental to understanding the full complexity of diseases (causal agent, animal, owner, environment, interactions between actors). The approach allows for data from the past and present of diseases and consider their possible future.

4.

I agree with the definitions, but I believe that PE can also be used to gather quantitative data.

5.

Well, to me PE should be redefined by combining the previous tow definitions of Mariner, J.C. and Paskin, R. (2000) and Catley, A.; Alders R.G., Wood, J.L.N. (2012)

I would define PE as, "Participatory epidemiology is the systematic use of participatory approaches and methods to understand about disease situations (could be occurrence, control and prevention practices, impact etc) with active involvement of community observations, traditions and existing veterinary knowledge." The term 'participatory' refer to the active involvement of communities incorporating existing traditional knowledge while Epidemiology (Epizootology?) refers to the science about incidence, distribution, and control of disease in a (animal) population. So, I suggest modification of the definition to be more comprehensive

and specific to Epidemiology. If it is about participatory technique, then the definition should be different.

6.

The two definitions cover the most important features of PE:

- First, acknowledgement that there is epidemiological data/intelligence held by livestock keepers;
- That this data is mostly qualitative;
- And that the interaction with and contribution of livestock keepers must be truly participatory and extend to all stages of the exercise – planning, implementation, data analysis, feedback, etc.;
- Would be good to add a sentence somewhere about how data collected can be analysed in conjunction with the more traditional quantitative data to give an enhanced understanding of epidemiology;
- After all, epidemiology goes beyond data collection. Methods of analysis and interpretation are just as important;
- I definitely agree that as an emerging discipline, examples of PE are essential to clarify and reinforce the definition and limits of the discipline.

7.

For me, definition issues don't weight heavy (possibly due to my limited English language skills) and I am more interested in the approach and application. With regard to Mariner's definition, my only suggestion is to leave out the 'emerging field' part. Already now, let alone in five years time that is no longer relevant. Of the two, my preference is with Catley as it less stresses the community level – implicitly referring to the level of households/livestock owners. I think participatory epidemiology can also be used for improving the understanding of diseases and options for disease control when dealing with other than communities, such as central and peripheral veterinary authorities, or private stakeholders such as farm managers. In my opinion, key to participatory epidemiology is the two-way direction of collecting information and developing disease control options as Catley is referring to when stating "therefore should go beyond the simple provision of information to outsiders". My experience is limited to working with veterinary authorities in Asia and Africa and I would argue that even with them using participatory epidemiology is relevant. More often than not, quantitative data (on disease incidence, on livestock and livestock owners populations, on value-chains) are absent, while they ask for revising or developing disease control plans. We, then use more or less similar techniques as in PE to retrieve information and data. Thus, in both definitions the word 'communities' maybe too restrictive if it refers to households, farmer communities or other field level communities. It is equally possible that my English language skills do not suffice for getting involved on definitions (as mentioned above).

8.

I will not be available for discussion on Wednesday but would be happy to follow. IN my experience the second definition by Catley ed al, is more complete. Participatory epidemiology does not only encompass the use of participatory techniques to harvest qualitative information, but also for ensuring acceptability, trust, sense of ownership into for instance an animal disease surveillance system.

9.

I agree more with the second definition, as I have discovered that in most occasions and locations (Africa and Europe) where I have used PE, farmers often don't even know that practices on their farm is/are the major insight in to understanding the disease situation in question for sustainable control. Furthermore PE also provided researchers avenues to be in touch with reality on ground so that research can be both action- and people- oriented. And at times PE helps researchers to understand why their predicted findings based on assumed hypothesis are not in tune with reality in the field. Essentially, PE is solely aimed to improve communal understanding of disease situation for its sustainable control. In this context, communal implies that everyone (i.e. community members and outsiders/researchers) involved in the exercise are ready to cooperate, such that knowledge gained or sourced is collectively processed and utilized by all parties involved thus ensuring the sustainability of the end result, which is disease control. In effect, PE is relevant to all parties (community members and outsider-the researchers), but it must be designed such that the community members sees the exercise as theirs for ownership role which in turn ensures sustainability.

10.
There are slightly different terms – but I think for the much of the same concepts – used by NIH and CDC in the US:

- Community based participatory research
- Community enabled participatory research

These terms are more "bottom up" than the terms you provided, which focus on the epidemiologist and her/his methods. The NIH/CDC terms focus directly on the community and differ in the extent of community involvement and autonomy. Note that a community can be variously defined – for example, in our studies of zoonotic pathogen exposures for farm and slaughterhouse workers, the community is the workforce.

11.
I agree for the 2 definitions, but we need to highlight that many important zoonotic diseases are concerned. Suggestion: The 2 definitions to be combined and read as follows, "Participatory epidemiology is an emerging field based on the use of systematic participatory techniques in harvesting qualitative epidemiological intelligence contained within community observations, existing veterinary knowledge and traditional oral history to improve understanding of diseases and options for animal disease control."

12.
The first definition is too narrow: PE can also be used for quantitative data such as cases, numbers of animals, dates. Also data collected using qualitative methods can be analysed quantitatively. I find "harvested" a rather extractive term: a key principle of participatory methods is that communities and researchers develop information together – it is not a question of 'harvesting'. The first definition does not define 'participatory' or 'epidemiology'
The second definition is broader and defines participatory well. The epidemiology definition is less correct as epidemiology is the study of health and disease in populations. The definition

could cover participatory methods used for diagnosis or clinical treatment. Also "options for animal disease control" is too narrow as PE is also used to evaluate disease control.

13.

I am more sympathetic with definition provided by Catley et al., as it enforces the concept of collecting data to react (and possibly change) the present sanitary situation making best use of the information and feedback from the beneficiaries of your future likely interventions. In the veterinary sector, this becomes fundamental when working in the sphere of zoonotic diseases (but not exclusively). For example, participatory epidemiology in project focused on Rabies (I was involved in) had the advantage of combining the gathering of epi info at village level, discuss the preliminary findings with communities and provide immediate feedback on the precautionary/prevention measures to be taken in case of the identified hazardous behaviors by communities and/or rabies suspects/emergency.

14.

In my opinion, both definitions are good enough. In the first one, maybe "an emerging field" is currently unnecessary. I realize the first definition as very comprehensive and easy to understand for people not deeply involve on epidemiology. The second definition is synthetic and could depends on additional explanation for people not familiarized with this approaches. However, rather than improve them a merged definition could be useful. I would suggest "Participatory epidemiology is the systematic use of participatory approaches and methods for the harvesting of qualitative epidemiological intelligence contained within community observations, existing veterinary knowledge and traditional oral history to improve understanding of diseases and options for animal disease control."

15.

I agree to the two definitions. They capture the subject of PE satisfactorily.

16.

As we are trying to promote one health approaches and get PE adapted in public health emergencies, the 2 definitions can be improved in to one like, 'participatory epidemiology is an emerging field that systematically uses participatory techniques to improve understanding of diseases within communities and options for disease control.'

17.

I agree with both definitions. However, the first definition can be improved by adding quantitative Epidemiological Intelligence. We should note that some tools and methods used in PE can actually capture quantitative data.

18.

I agree with PE being an active way of communities understanding their problems and I think it should be moved forward to dealing with the problems as well. PE I think was initially coined by animal scientists but a one health approach seems to be more attractive in modern times.

19.

I tend to agree more with Mariner and Paskin's definition. I have used PE techniques in evaluating the constraints faced by nomads and other underserved communities when accessing routine immunisation services for eligible children and got fascinating results. PE should not be restricted to animal diseases alone, vets are now playing key roles in one health and hence increasingly using their skills in solving human health related issues. The definition should be broad to capture this.

20.

Participatory Epidemiology Is a tool used for disease surveillance through harnessing traditional knowledge by using participatory approaches and methods to improve our understanding of the patterns of disease in populations. Participatory epidemiology was first developed in pastoralist systems for rinderpest active surveillance, PE is now applied in a variety of livestock systems to endemic, epidemic and emerging diseases. Participatory epidemiology is based on communication and transfer of knowledge, using a variety of tools:

- Informal interviewing: semi-structured interviews, with key informants, focus-group discussions
- Ranking and scoring: simple ranking, pair-wise ranking, proportional piling, matrix scoring
- Visualisation: mapping, timelines, seasonal calendars, venn diagrams

Participatory Epidemiology Methods are complemented by secondary information sources, direct observation, and laboratory diagnostics. Data is cross-checked through probing and triangulation.

21.

The first definition (Jeff's) seems to limit PE to qualitative methods. But we know that quantitative techniques can be also be applicable. The qualitative data can be transformed into quantifiable variables which can then be analysed quantitatively. This is rarely the case but is a possibility. So I would not limit PE to qualitative approaches alone.I also have reservations about the word "emerging". When shall we decide that PE has now emerged? The second definition (Andy's) appears more comprehensive. However, I would add the words "....options for disease surveillance and control" at the end. This is in recognition of the utility of PE in routine surveillance.

22.

Things evolve over time - while the first definition was applicable in 2000 a lot of happened since then. I think that the 2nd definition is better more inclusive. In 2009 we met with a group

of PE/PDS trainers at a PENAPH training of trainers on PE came up with the following principles:

- PE is an approach to epidemiology including active surveillance that is sensitive to and benefits the community conducted by professionals.
- It is an interactive dialogue conducted within the community, combining scientific and traditional information with the aid of Participatory Rural Appraisal (PRA) tools that allows for discovery by interviewer and community.
- It is flexible, semi-structured and adaptable to changing situations. Data from multiple sources is rapidly analysed for quick feedback and response.
- It is founded on equal partnership with mutual respect and trust encouraging positive attitude to enable community empowerment.

I think that the bullets include some key issues that the 2 definitions don't have:

- The fact that it is done by professionals and not by simple enumerators
- The last bullet is also not addressed in the 2 definitions you shared.

23.

An on the spur definition: Participatory Epidemiology is the application of participatory approaches on community-based stakeholders in order to identify, prevent and control community based diseases and other well-being problems.

24.

Limiting the definition to understanding "animal disease control" or "existing veterinary knowledge" could imply that PE has no place in human health, which is not true. I would use the word "health" as a substitute to these two phrases quoted. I have practical experience applying these techniques to unique human settings. One research eventually led to an appropriate intervention in the affected community. Mothers in this community were eventually offered an operating theatre to reduce maternal deaths in their community!

25.

Would propose the following (from the second definition): "Participatory epidemiology is the systematic use of approaches and methods to improve understanding of diseases and options for animal disease control." I delete "Participatory" as this cannot be used in all the cases.

26.

About PE definition: the 2 definitions sound a bit different to me in terms of community empowerment, and they induce different postures for researchers and field operators involved in PE actions. The first one seems to be more focusing on knowledge sharing, data collection, tools taking into account "traditional" health perceptions/representation and local/diverse points of view about animal diseases' management. There is no explicit reference to action taken by the communities. Maybe because this knowledge is implicitly collected in order to be used in the framework of conventional surveillance systems and will be managed and highlighted by Vets and animal health specialists. The second one is more opening a pathway toward collective action and seems more oriented on the research of solutions and alternatives to conventional control strategies. Being implicit that local communities will be empowered in this process to take decision in order to better manage animal health problems (not a priori under the authority of vets or animal health specialist). Maybe both definition are

complementary and correspond to different phases in the PE process (knowledge sharing/decision making).

27.

From my perspective, I think that it can be unproductive to get overly hung up on semantics and spend too much time arguing about definititions and I often tell my graduate students to clearly state the definition that they use and then use it consistently. That said, clarity is indeed important. I think the Mariner and Paskin (2000) definition is useful but in 2015 perhaps time to drop the word ‘emerging’ as it is a well-established and credible field of research. I also find it limiting in the use of ‘qualitative’. My own definition would also include a citizen science element as well – in some cases participatory epidemiology might include hunters collecting biological samples for lab analysis. I prefer ‘experience-based knowledge’ to traditional oral history because some information is collected through acquired knowledge from others and some is collected from a person’s own experiences and observations.

28.

Yes PE uses participatory approaches in understanding disease of interest and through focus group discussions disease mortality and other variable can be estimated, temporal occurrence of disease through seasonal. Also awareness and clinical recognition of diseases and control/prevention options can be assessed.

29.

Using participatory technique/approaches to know from community what local diseases affect humans, animals and plants with possible knowledge of causative agent and remedy to control them in their local environment. This is based on my field experiences.

30.

I totally agree with both definitions. But in my opinion neither is complete because of no taking into account the evolution of participatory epidemiology. For someone like me who worked in PDS I find myself in the second definition. But other one who made participatory disease searching could agree with the first definition. So I offer this definition: "Participatory epidemiology is the systematic use of participatory approaches and methods to improve understanding of animal diseases searching and surveillance."

31.

I agree with the two definitions

32.

Reading through those two definitions, the second one by Catley et al. seems to be closer to my understanding of the PE-approach. Mariner et al seem to be limited to the idea of “harvesting” without really involving the community at the level of project development and control strategies, as is stated in the second paragraph of Catley et al. I’m also not sure about the sole focus on “qualitative epidemiological intelligence”, since there might also be semi-quantitative intelligence coming out of such a project. One thing I’m missing in both definitions

is the One-Health approach, considering the fact that quite a few livestock diseases, or EIDs that might affect farm animals, are zoonotic or have a zoonotic potential. Catley just speaks of "animal disease" and Mariner mentions veterinary but not physician knowledge. Included in the One-Health idea would also be recording and follow-up if there is salience of disease dynamics in wildlife populations in proximity of livestock.

33.

I think that the first definition is better explain what PE is all about. When you say 'improved' what do you mean? I would not be keen to make changes as I believe is a sort of "official" definition. "Participatory epidemiology is an emerging field that is based on the use of participatory techniques for the harvesting of qualitative epidemiological intelligence contained within community observations, existing veterinary knowledge and traditional oral history."

"Participatory epidemiology is the systematic use of participatory approaches and methods to improve understanding of diseases and options for animal disease control." The term 'participatory' should be used to refer to the active involvement of communities in the definition of project objectives and development of disease control strategies and therefore should go beyond the simple provision of information to outsiders.

34.

"Participatory epidemiology is an <u>evolving</u> branch of veterinary epidemiology based on the active participation of local communities (livestock keepers, pastoralists, farmers, village elders, and other relevant stakeholders) and local experts (key informants such as veterinarians, wildlife biologists, land use planners, decision makers, etc.) in sharing indigenous and expert knowledge on animal health and production as well as defining and prioritizing project objectives and development of disease control strategies in order to improve understanding of diseases, and options for animal disease control. PE integrates the systematic use of participatory approaches and methods for data acquisition (e.g., informal interviews, visualization/mapping and ranking/scoring methods) with standard veterinary investigation procedures and qualitative and quantitative statistical analysis".

35.

I am not sure whether participatory epidemiology is only used at community level – if this is the case, then this should be added in the second definition. The two definitions refer to participatory techniques or methods or approaches – which is the most correct term?

36.

I agree with both definitions. I think Jeff's definition was developed earlier and is perhaps aimed to Africa and nomad circumstances. In America, we don't have such a millenary knowledge regarding livestock keeping, however PE is very useful in acquiring current knowledge. Andy's definition is more complete, including the intention for such knowledge acquisition (control) and states that participation of community is NOT merely as data provider. On the other hand, I think overlooks the (by not mentioning) the circumstances of the community, despite along his paper it is clearly included.

37.

To me the first definition seems to be lacking clarity as to what use the epidemiological intelligence so gathered will be put. It is also my feeling that the aspect of safeguarding international trade be incorporated in the definition of PE, especially with regards to its use in risk analysis.

38.

I will go for the definition provided by Mariner and colleagues as being more comprehensive. Whereas the other one, by Catley , seems to limit PE only to disease control. To my understanding PE has a much broader role as indicated in the following definition by Mariner. Participatory epidemiology is an emerging field that is based on the use of participatory techniques for the harvesting of qualitative epidemiological intelligence contained within community observations, existing veterinary knowledge and traditional oral history." It is my personal experience that for working, in a traditional agrarian or semi agrarian society, on any initiative pertaining to livestock, harvesting of community inspirations, which are largely based on the observations and traditional knowledge, and using this information to devise the relevant strategy is the key to success. Such strategy will incorporate the inspirations of the people and will earn the owner ship by the people.

39.

The second definition provides a well description of PE including the definition of the term "participatory" and also refers to the "active involvement of communities".

40.

"Participatory Epidemiology is a real time epidemiologic approach that actively involves end user/beneficiaries of disease control interventions in a specific locality; it entails an interactive gathering of all information deemed relevant to the event under investigation, designing and implementation of intervention strategies. It obviously builds pragmatic consensus among experts and communities at all level of event investigation and containment thus it potentially guarantees success and sustainability through the experts' better understanding of local context as well as the ownership by communities of every bit of the approach".

41.

I would go more with Catley, A, et al. concerning the definition of PE but with some slight modification: "Participatory epidemiology is the systematic use of participatory approaches and methods for better understanding of diseases and its control through active involvement of the community"

42.

"Participatory epidemiology is an emerging field that is based on the use of participatory techniques for the harvesting of qualitative epidemiological intelligence contained within community observations, existing veterinary knowledge and traditional oral history."

This definition is somewhat narrow in scope. The aim of the participatory epidemiology is not only to apply techniques involving data acquiring from the community members. It is to

involve the community in the research, disease control or disaster risk reduction projects as active subjects, not only as the object of observation. In order to address the vulnerabilities of the community and improve its resilience in a sustainable manner, we need the active participation of communities. Harvesting epidemiological intelligence is only one facet of the participatory approach. Second, why harvesting only qualitative data? There were successful projects with quantitative data acquired by i.e. SMS data submission (eg. Philippines). The veracity and validity of the data may be questionable, but it applies to both qualitative and qualitative data.One of the similar definitions:

- Schwab M, Syme SL. On paradigms, community participation and the future of public health. Am J Public Health 1997;87:2049–52.):
  (participatory epidemiology) "implies working across disciplines and with the population itself, in defining variables, designing instruments, and collecting data (qualitative and quantitative) that reflect the ecological reality of life in that population, as people experience it."
- Catley., A; Alders RG, Wood JLN (2012). Participatory epidemiology: approaches, methods, experiences. The Veterinary Journal, 191: 151 - 160

"Participatory epidemiology is the systematic use of participatory approaches and methods to improve understanding of diseases and options for animal disease control." The term 'participatory' should be used to refer to the active involvement of communities in the definition of project objectives and development of disease control strategies and therefore should go beyond the simple provision of information to outsiders. This definition is broader in scope and more open. It stresses the active involvement of communities, which actually empowers their members, aside from being more willing to take part in a project. Any definition requires an established context; and, the context is not epidemiology, nor the research, but populations in their natural and social environment. There are numerous definitions of participatory epidemiology, and a good overview can be obtained in Margaret W Leung, Irene H Yen and Meredith Minkler Community based participatory research: a promising approach for increasing epidemiology's relevance in the 21st century, Int. J. Epidemiol. (2004) 33 (3): 499-506. This citation points well to the essence of participatory epidemiology: "The application of the principles of Community Based Participatory Research in such studies provides guidance for epidemiologists who wish to use their skills and training to advance health promotion and disease prevention with and for the public rather than on the public."

43.

I agree with the definitions but think that it could be emphasised that PE is an iterative process involving both the collection of data and knowledge, the exchange of knowledge, feed-back and follow-up to develop strategies/solutions together.

44.

The second one by Catley and Wood seems to be more appropriate

45.

I agree with both definitions, but the definition of Catley, A.; RG Alders, JLN Wood (2012) will might be more accurate. To this definition I would like to add as follow "Participatory epidemiology is the systematic use of Participatory Approaches and Methods to improve understanding of diseases and options for the animal disease prevention and control, to improve the health of the Communities.

46.

I especially agree that PE is both qualitative and quantitative and as such, no need to have either word in the definition. Perhaps adding something on improving the involvement of communities in the analysis of animal disease problems and the design, implementation and evaluation of disease control programmes and policies

47.

I would agree with Catley's definition on PE because the term of participatory involve multi elements in the community.

48.

I am surprised that we put up a definition from 1990, but perhaps it was the first. PE has certainly evolved from that first book proposing the approach as an emerging field.

I agree with the comments that ‘emerging’ is no longer appropriate. The approach is in fact institutionalized at many levels. It’s included in some textbooks and in regular curricula of Universities in both developing and developed countries. It is also appears as a topic in international meetings and is utilized by a wide selection of countries and international organizations. I can support the removal of ‘qualitative’ but think we need to emphasize that PE is a learning methodology more than a research methodology. There are at least two very well written documents that describe the hazards of using participatory approaches to do quantitative research and I think some examples of ‘quantitative uses of participatory approaches’ actually sacrificed their participatory nature. The issue is that as we move to standardize questions and make the process more amenable to quantitative analysis we lose the special value of the participatory approach which is to empower people to express their own ideas in their own words and to encourage learning for all involved. If you go to the resource page of the PENAPH website you will find the two documents I referring to. Also, one point that has always nagged me since I wrote the PE Manual is the principle I was thought in grade school that you can’t use the word in its own definition as it becomes a circular statement. Many of the definitions boil down to participatory epidemiology is the use of participation techniques in epidemiology. This is not very helpful for those who have been exposed to the complex topics of participation or epidemiology. Picking up where you helpful edits left off, I would suggest the following definition: “Participatory epidemiology uses participatory techniques to create a shared learning environment leading to a better understanding of the epidemiological information and knowledge contained within community observations and experience-based knowledge to inform joint decision-making and action.”

Participation is the empowerment of people to identify and solve their own problems.

Epidemiology is the study of the patterns of disease in populations in order to understand their impact, cause and identify actions that can mitigate their impact. In recent years, my involvement with PE has focused more on One Health applications. The tools are well suited to looking at complex eco-health issues and solutions such as the problem of Ophisthorcus in Southeast Asia

49.

I delayed responding to this issue because clearly I'm partial to the definition included in the Catley et al. paper. The importance of participatory approaches and functional participation of all key stakeholders is essential in terms of making real progress in relation to animal health and production and wider ecohealth issues.

50.

The use of the word "participatory" in the definition results in a circular argument. An alternative word or phrase for "participatory" in the definition would be preferable.

51.

In my opinion, the definition of Catley et al describes the term in the best way. I think it is difficult to explain the term of participatory epidemiology by using the word "participatory methods". I think it is important, that the definition includes a definition of the term participatory as well.

52.

I tend to favour the Catley (2012) definition, and it is mostly because it provides a definition or clarification on the term participatory. I think a preferable definition of PE would not use the words "participatory" or "epidemiology" (or some derivative thereof) in the definition. It leads the definition into circular reasoning that is not clear. You could say that all epidemiology involves participation by communities in the gathering of data. I think the distinguishing feature of PE is the active involvement of the "communities" in the project goals and development of options. The project, is, in a sense, "owned" by the participants AND the researcher as opposed to being "owned" ONLY by the researcher (as in the case of non-PE epi…or do I now call it traditional epi?). "Participatory epidemiology, in animal health, is the systematic use of approaches and methods—using the active involvement of communities (i.e. all stakeholders involved) in the definition of project objectives and development of disease control strategies—to improve understanding of diseases and options for animal disease control." Communities might include those who own/raise/treat/butcher/etc animals in the geographic area of interest. It might provide clarity to the definition to provide some examples of what a "community" might be, although that could certainly change with the situation.

53.

Catley et al., with modifications is preferred. I think the link between Ecohealth and participatory epidemiology is missing. Participatory epidemiology is integrated in the heart of an EcoHealth approach and active community involvement. So it's not just about diseases, but about community mobilisation to address public health challenges (so you can use

participatory epidemiology to assess health risk link with pollution, farm management, waste management, contact with wildlife population, loss of biodiversity, etc). Moreover participatory epidemiology is actually seen as a very important tool to use for the evaluation of OH actions and impacts (see NEOH Cost action: http://neoh.onehealthglobal.net/). I think as well that in participatory epidemiology the concept of interactive mutual learning process should present as during practice of participatory epidemiology knowledge sharing is more effective. Another point, Participatory epidemiology can as well help to have a better understanding of farmers/stakeholder perceptions about a risk or an health problems, not only assessing knowledge, and we know that it's important to understand attitudes and reactions when facing health challenges and control strategies that are provided by external stakeholders.

54.

The definition is rather circular, "use" and "using" are repetitive – it does need to be "systematic" in fact some studies utilize participatory methods in part but not in whole "Participatory epidemiology is the incorporation of systematic use of ~~participatory~~ approaches and methods that actively involve the community or group being studied using the active involvement of communities (i.e. all?? Really? I have rarely seen an industry or pollution source involved! stakeholders involved) in the definition of project objectives, development of interventions including exposure control and treatment~~disease~~ strategies, and to improve understanding of health risks ~~diseases~~ and options for ~~disease~~ surveillance, control, and health evaluation in populations NOT SURE ABOUT THIS LAST PART; SEEMS TO BE REPETITIVE AND ALSO UNDULY RESTRICT THE GOALS OF EPIDEMIOLOGICAL STUDIES.."

55.

I think the PE definition provided by Catley seems broder , but it has also its limitation. It is only focused on animal disease control which I consider is very narrow and suggest to be replaced by the current one health thinking " animal and human disease control" including the ecology. You can choose the best combination of words that may reflect zoonosis & antropo-zoonosis control & eradication strategies. The phrase "provision of information to outsiders" is disappointing, because local researchers are also working on PE therefore , I suggest to be removed from the definition.

56.

I think the discussion has been very valuable, but I don't think replacing 'participation' with 'active involvement' is adequate. It would be better to provide a clear definition of participation as part of the definition. Its a term that is frequently misunderstood by those new to the area and appropriate participatory practice is at the heart of doing good PE.

57.

I was unfortunately not able to respond to the discussion last week. I do agree with the changes to the definition. The more I look at it and am exposed to other professions I have realized that they all use similar approaches for community participation but call it something different. For example, community-based participatory approaches (CBPR) from nursing, or Ecosystem approaches to health (AMESH, etc)...and the list could go on from here. The question that keeps coming to my mind is....what makes participatory epidemiology different that these? I keep coming back to the tool set that participatory epidemiology uses in its process. In the recent course that my graduate student and I took from Jeff Mariner, we learned participatory methods such as transect walks, focus group meetings with non-structured interview techniques, calendars or proportional piling and matrix building (bean exercises) to engage groups discussion and permit translation of community knowledge into semi-quantitative data, outbreak investigation through both historical and acquired knowledge of the community, etc. None of the definitions included the uniqueness of the defined set of activities that seem to me to set apart the PE approach from other discipline approaches that I have encountered. My graduate student's project involves working with interested First Nations and Metis communities in Saskatchewan Canada to assess dog population and dog bite issues. The community's defined their issues, defined the control programs that they would initiate, and we have worked with them to document the changes in quantitative methods (dog counts, etc) but also have documented the community process and struggles to work on these issues within the context of other competing community health issues. In describing her project for a poster for the upcoming conference called ISVEE 2015 in Mexico, we called it "community-based participatory approach using participatory methods such as transect walks, venn diagrams, non-structured interviews, ranking, proportional piling and matrix building". This decision comes because the other members of the student's graduate committee are either from nursing or ecohealth circles where PE is not a well-known or well-used terminology. Most of the examples in week one appeared to involve use of these methods in developing countries. My question would be whether the term PE is only used there because we are trying to differentiate "yet another method" from already established methods of community-based research used in developed countries? This question does not just relate to PE as I am having the same discussion on what differentiates "Citizen science" from CBPR or PE.

58.

I think the definition as below is still problematic: "Communities" are not synonymous with "all stakeholders" involved. It is possible to do PE with groups of people which would not fit the definition of a community without 'participatory' in the definition there is not way to distinguish this from other forms of assessment. The reference to 'setting project objectives' does not seem to fit and assumes there is a project. It is a little confusing to talk of health risks as we often refer to diseases risks.

Comments related with PE applications

1.

We used the approach in the southern district of Chikwawa here in Malawi when we were investigating an outbreak of Foot and Mouth Disease in cattle. The approach is great to say the least. We were faced with this outbreak soon after our staff were trained in participatory epidemiology by Dr Saskia Hendrikx of ILRI courtesy of USDA/APHIS.

2.

We have used participatory epidemiology throughout our village poultry health and production and food and nutrition security research and development. The participatory epidemiology and participatory methodologies we use are documented in:

- Ahlers C., Alders R.G., Bagnol B., Cambaza A.B., Harun M., Mgomezulu R., Msami H., Pym B., Wegener P., Wethli E. and Young M. 2009. Improving village chicken production: a manual for field workers and trainers. ACIAR Monograph No. 139, pp. 157-172. Available: http://aciar.gov.au/publication/mn139
- Alders, R., Aongola, A., Bagnol, B., de Bruyn, J., Kimboka, S., Kock, R., Li, M., Maulaga, W., McConchie, R., Mor, S., Msami, H., Mulenga, F., Mwala, M., Mwale, S., Rushton, J., Simpson, J., Victor, R., Yongolo, C. and Young, M. 2014. Using a One Health approach to promote food and nutrition security in Tanzania and Zambia. Planet@Risk (Special Issue on One Health) 2(3):187-190.
- Alders, R.G. and Spradbrow, P.B. 2001. Controlling Newcastle Disease in Village Chickens: a field manual. Canberra. Australian Centre for International Agricultural Research (ACIAR) Monograph 82, pp. 56-60. Available: http://aciar.gov.au/publication/mn082
- Azhar, M., Lubis, A.S., Siregar, E.S., Alders, R.G., Brum, E., McGrane, J., Morgan, I. and Roedar, P. 2010. Participatory Disease Surveillance and Response in Indonesia: Strengthening Veterinary Services and Empowering Communities to Prevent and Control Highly Pathogenic Avian Influenza. Avian Diseases 54(s1):749-753.
- Bagnol, B., Alders, R.G., Costa, R., Lauchande, C., Monteiro, J., Msami, H., Mgomezulu, R., Zandamela, A. and Young, M. 2013. Contributing factors for successful vaccination campaigns against Newcastle disease. Livestock Research for Rural Development Volume 25, Article #95. Available: http://www.lrrd.org/lrrd25/6/bagn2506cit.htm
- Further examples of PE are available at: http://www.participatoryepidemiology.info/

3.

I have conducted my research for my PhD ON "INTEGRATING PARTICIPATORY EPIDEMIOLOGY FOR DEVELOPING CONTROL STRATEGIES FOR TRANS-BOUNDARY ANIMAL DISEASES IN SINDH PROVINCE OF PAKISTAN" with special reference to FMD & PPR
Previously animal disease control strategies were developed in Sindh Province of Pakistan based on Field disease reports and serology and these have not yielded desirable results. Based on the findings of my research when information derived through PE was integrated with Field disease reports and serology to develop disease control strategies, this has been very successful because observations of the community were taken into account.

4.

I have a good example of PE that fits the modification of Catley et al. WE developed a model which was already validated to be suitable across Europe. However, it didn't work well for almost all farms in the Campania region of Italy. So I had to travel down there to conducted a PE exercise. On arrival, I discovered the veterinarians and farmers have been wondering why the disease does not follow the usual expected seasonal pattern. The PE exercise revealed that the movement of sheep for grazing (by farmers) based on the dynamics of the climate in the region is responsible for the peculiar distribution/pattern of the disease amongst sheep. In effect, the PE exercise helped me to better validate and appreciate the limitation of my model and to explain how host movement is critical to sustainable control of the disease. Additionally the farmers and vets got the answer they have been seeking for, by so doing, they have come to appreciate the significance and how the farmer's practice of host movement based on changes in climate predetermines the real-time seasonality of the disease in question on the farm (PE revealed what was right before their eyes all along).

5.

Participatory assessment of RVF in northern Tanzania(2006/7). The value accrued from such studies: RVF within Maasai community was not clearly known despite of immense knowledge of animals diseases. Translating disease syndrome('orikibrito") meaning 'abortion' we manage to understand better the disease condition. Communities have enormous disease knowledge often expert are not aware of!

6.

I experienced PE approach during a project aimed to investigate the epidemiology of AI in Moeyingyi wetland area (Myanmar, 2009) where we used semi structured interviews, seasonal calendars and other PE tools, but due to donor, budget and time constrains it was difficult to ensure “the active involvement of communities (i.e. all stakeholders involved) in the definition of project objectives”. A possible improvement would have been to enlarge the number of stakeholders involved during the feasibility/inception phases, to improve budget allocation, to find additional or different donors.

7.

Although I am big fan of PE techniques but have faced two main problems. Monitoring work of PE workers due to flexibility nature of PE methodology. I mean there are some black sheep every where in the world who create data by sitting in office without visiting or involving communities. So I suggested Some fixed Checks for monitoring. Presenting enormous data we obtained during PE activities.I mean simple suggestion for Statistical analysis as we carried out with traditional Epidemiology data. So I suggested we need to finalize statistical analysis tools not only for presentation but also give understanding for those epidemiologists who do not have complete understanding of PE.

8.

I think many KAP-studies are presented as PE, but according to the definition they are not as the participatory element is weak, especially then it comes to the actions to be undertaken to solve the problems discussed.

9.

I have applied PE to collect data for Economic evaluation of impact of rabies in livestock in Ethiopia.

10.

Regarding examples of PE activities in Ethiopia, currently the Ethiopian Ministry of Agriculture & Rural Development is conducting a USAID funded project entitled, "Assessment of the magnitude and causes of calf mortality in major production system in Ethiopia". This project is totally dependent on PE techniques to achieve its objectives. The field work was conducted from June 2015- September 2015, currently the collected information is under analyses, final report to be published in December, 2015. Also, the MSc research work published by Rufael et al. (2008), entitled Participatory epidemiology as compared to conventional foot and mouth disease surveillance tool conducted in Borena pastoral areas of Ethiopia has identified the relevance of indigenous community knowledge on FMD detection and diagnosis compared to conventional disease surveillance tools. I think this work may be a good example of PE activities.

11.

Study on the development of a participatory assessment tool on the system of Community Animal health workers in Cambodia. The participatory approach has been used to take into account the different perspectives of the concerned actors, but also for the purpose of ownership of the tool.

Calba, C., Ponsich, A., Nam, S., Collineau, L., Min, S., Thonnat, J., Goutard, F.L., 2014. Development of a participatory tool for the evaluation of Village Animal Health Workers in Cambodia. Acta Tropica 134, 17–28. doi:10.1016/j.actatropica.2014.02.013

Studies on the evaluation of the costs and social benefits of health surveillance in the Viet Nam and Thailand; combining participatory approaches, economic assessment tools.

Delabouglise A, Antoine-Moussiaux N, Phan TD, Dao DC, Nguyen TT, Truong DB, Nguyen XNT, Vu DT, Nguyen VK, Le TH, Salem G, Peyre M. 2015a. The perceived value of passive animal health surveillance: The case of Highly Pathogenic Avian Influenza in Vietnam. Zoonosis and Public Health (online ahead of print version, April 2015)

Alexis Delabouglise, Nicolas Antoine-Moussiaux, Dumrongsak Tatong, Arun Chumkaeo, Aurélie Binot, Guillaume Fournié, Eva Pilot, Waraphon Phimpraphi, Suwicha Kasemsuwan, Mathilde Paul, Raphael Duboz, Gerard Salem and Marisa Peyre. Cultural practices shaping zoonoses surveillance: the case of Highly Pathogenic Avian Influenza and Thailand native chicken farmers. Submitted to EcoHealth

Use of participatory epidemiology to analyze the subjectivity of individuals facing a health situation and identify connections and trends in opinions within different social groups.

L. Chiffot, N. Antoine-Moussiaux, S. Boutmao, S. Morand, J. Cappelle, A. Tarantola, F. Goutard. (2014) Participatory methods to explore rodents-related health risks perception among rural farmers of Cambodia. P2.13- Poster Presentation at 5th Biennial Eco Health Conference, Montreal, Canada

Studies on the organization of surveillance and animal production networks, combining participatory epidemiology and social network analysis.

Baudon E, Fournié G, Dao TH, Pham TTH, Duboz R, Gely M, Peiris M, Cowling BJ, Ton VD, Peyre M. Analysis of swine movements in a province in Northern Vietnam and application in the design of surveillance strategies for infectious diseases. Transboundary and Emerging Diseases 2015 Jun 4. doi: 10.1111/tbed.12380.

A Delabouglise, T H Dao, D B Truong, TT Nguyen, N T X Nguyen, R Duboz, G Fournié, N Antoine-Moussiaux, V Grosbois, D T Vu, V K Nguyen, T H Le, G Salem, M Peyre. When private actors matter: information-sharing and surveillance of Highly Pathogenic Avian Influenza in Vietnam. Acta Tropica 2015 Jul;147:38-44. doi: 10.1016/j.actatropica.2015.03.025. Epub 2015 Apr 3.

12.

Participatory methods have been successfully used for the reason of finetuning surveillance activities and for the evaluation of surveillance systems. Some examples are the use of AccEPT, a participatory scoring method, in European animal health surveillance programmes to assess and discuss the process of the system itself, the trust in the system and the acceptability of the system by the full range of its stakeholders (from all levels); or the use of SWOT analysis (strength weaknesses opportunities and threats) to ensure every stakeholder has a say and priorities for amendments of a system are made jointly (each stakeholder provides a SWOT of the system from their point of reference, the weaknesses and room for improvement are prioritised in a round table discussion and adaptations to the process of the system that can be made within short time effectuated). Those methods could equally be applied for joint decision making and prioritisation of activities, i.e. referring to the second sentence of the definition by Catley et al.

13.

For example, participatory epidemiology in project focused on Rabies (I was involved in) had the advantage of combining the gathering of epi info at village level, discuss the preliminary findings with communities and provide immediate feedback on the precautionary/prevention measures to be taken in case of the identified hazardous behaviors by communities and/or rabies suspects/emergency.

14.

After May 2009 training on use and application of PE in animal disease surveillance in Arusha-Tanzania, we were given funds to go for practical use of PE in disease surveillance. We managed to access the efficiency and sensitivity of the tool by doing surveillance in areas which we were sure of diseases status surprising enough the results we obtained was not very fur from what was known previous.

The technique also was used on the outbreak of unknown condition on Maraboo storks in Kagera Region in Tanzania. Generally it was realized that PE as a techinique though it is expensive but has ability of capturing unpredicted disease so it is very easy even to come up with emerging and reemerging diseases something which is very difficult with other traditional technique.

15.
The surveillance program for African Swine Fever and Classical Swine Fever in Cape Verde is a good example of PE, where the community is actively involved in meetings and activities promoted by the Food and Agriculture Ministry.

16.
In recent years, my involvement with PE has focused more on One Health applications. The tools are well suited to looking at complex eco-health issues and solutions such as the problem of Ophisthorcus in Southeast Asia.

Comments related with the provided examples

1.
As for question 3 I do not feel comfortable to say questionnaire survey (structured or semi-structured). So e.gs. 1 and 2 cannot be considerd as PE.

2.
All these three examples are PE activities, Why? Because Participatory technique has been applied to gather the knowledge contained in the community.

3.
Example 1: The use of SSI involving the key people/and stakeholders makes this example a PE activity. Example 2: Not classical PE since data collection was done using questioners thus not participatory in nature. Example 3: Participatory because informal interviews are being conducted involving key persons and the veterinary services.

4.
I think only example 1 is an example of PE activity, in the other two samples in my opinion it is just an usual questionnaire, to gather information from different, also well informed people. However it does not include the opinion or the knowledge of people which are actually involved. So the people do not actively participate they are just answering pre formed questions and the outcomes will probably affected them just in a secondary way. In my opinion questionnaires are no participatory methods. In contrast I consider semi-structured interview as being one.

5.
Example 1 could be considered as a PE activity because all the approaches (semi-structure interviews, further investigation/triangulation and quantification/ranking) used are

participatory-based. Example 2 cant be considered as a PE activity as questionnaire is rigid and does not encourage active participation by all relevant stakeholders in the exercise.
Example 3 may in part be considered as a PE activity as the study employed informal interviews of key persons in the area of interest.

6.
For my understanding the examples 1 and 3 are representatives of PE approach.

7.
Within three examples you provided, PE is useful in example 1 and 3 because informal interview with open questions will allow gathering more and detail information. Ranking and scoring techniques can be used to collect quantitative data. In addition, PE can be applied in combination with other methods such as social network analysis (in example 3) to have better results. In example 2, it is not clear that they use structured or semi-structured questionnaire. To determine knowledge, attitudes, and practices, it is the best way to use PE with semi-structured interview because people will have a chance to express their opinion openly.

8.
Example 2 seems to defy the point of PE, because it's based on a questionnaire. Example 1 I see as a PE activity. The use of semi-structured interviews and the involvement of the different stakeholders in the bTB program, as well as the stepwise approach of gathering information to achieve a weighing of factors match my view on a PE project. The 3rd example I see as a fragment of a PE activity. I assume it's embedded in a more comprehensive project. The quite specific preselection of participants and the fact that the vet service is "officially involved" in an investigation of an, at least partially, illegal activity, might skew the information.

9.
With the three examples, it is difficult to say No, PE activity could not be considered. But when I'm referring to PE definition that I've chose (Catley et al., with modifications), I have some concern with the first example which is about social factors. I know that PE can be used in many fields, but used it to talk about social factors, I am not sure that it is a good way. For example2, I think that PE activities are suitable even if the target here is not animal but people. For example3 I also think that PE activities are suitable because it's about participatory risk analysis.

10.
Example 1: social factors influencing the eradication of Bovine Tuberculosis (bTB) in Spain: n this study PE tools was used which include semistructured interview with key informants, qualitative and quantitative data were obtained which are elements of PE. Example 3: Risk of introduction of Rift Valley fever (RVF) and foot and mouth disease (FMD) in Egypt through animal movements: in this project to describe animal movements pattern and estimate number of animals entering Egypt illegal and legal will need to have informal interviews which is one of the PE tool also data which will be generated will be qualitative and quantitative all those are elements of PE.

11.
I think that all 3 examples provided could be considered PE at different level of participation where example 1 seems the more participative one

12.
All three described studies seem to be lacking on the “participatory” part. Example 1 could be a PE-candidate as the issues to develop further were determined after the first round of interviews. Example 2 and 3 seem to have the objectives clearly set before conducting the work, so this weakens the PE-potential.

13.
Example 1 is Yes as they used semi structured interviews. Example 2 is No as questionnaires are not considered PE tools. Example 3 is Yes they informal interviews are considered a PE tool.

14.
Example 1: Yes.
- Has initially used the semi-structured interviews
- Involvement of different key stakeholders in the sector and
- Triangulation of the gathered information both in qualitative and quantitative appoach

Example 2: No, though I was unable to access the said fully in the website but am of the opinion that this did not conform with PE because:
- Seemed to have been conducted using questionnaires which are always straight and pre-determined
- Did not target several stakeholders rather only those who intended to participate in the Hajj or Umrah Muslim pilgrimages?

Example 3: YES.
- Involvement of different actors
- Study of the animal movement paterns based on the nature of the disease spread
- Use of the informal interviews

15.
I think they all fit PE in some way.

16.
Example 1: social factors influencing the eradication of Bovine Tuberculosis (bTB) in Spain: Yes, the tools used are related to participatory research.
Example 2: Knowledge, attitudes, and practices concerning Middle East respiratory syndrome among Umrah and Hajj pilgrims in Samsun, Turkey, 2015 (published in Eurosurveillance, Septembre 2015): No, not with the use of close questionnaire.

Example 3: Risk of introduction of Rift Valley fever (RVF) and foot and mouth disease (FMD) in Egypt through animal movements: Yes.

Topic 2

Comments related with how to incorporate PE activities within official surveillance systems, e.g., how to allow for laboratory diagnosis of PE findings, or how to combine with other (passive or active surveillance) approaches?

1.

To incorporate PE activities in mainstream surveillance activities, we need to develop a core mass of PE practitioners and experts through, for example, in-service training of epidemiologists, refresher courses, creating more access points/platforms for PE (a form of marketing) and designing-cum-incorporating PE in curricula for training epidemiologists.

2.

Specialized teams at regional/area level can be developed and PE should be used where its strength is. This methodology will have different roles in based on the development stage of the surveillance system of the country.

3.

Ensure that PE activities are handled and guided by veterinary professionals. This will facilitate iinstitutionalization of PE activities in the national surveillance systems of countries.

4.

PE as an important tool especially under certain circumstances (say remote rural settings with poorly educated population) where other method(s) may not get your wanted info or data should be advocated at regional and national levels. How such a tool will be important for detecting emerging conditions/diseases in a community and prompt for sampling for lab testing. So that countries can include that in their routine surveillance of even the curriculum in their relevant training institutions.

5.

Involvement of communities and animal stakeholders in disease surveillance is an essential factor of success and sustainability. The mainstream surveillance system should therefore pick upon any information coming from PE for verification through lab diagnoses and further assessment/investigation just like farmer disease does. Such complementary actions would encourage wide participation and collaboration, and greater effectiveness/efficiency of surveillance systems.

6.

A possible way to institutionalise PE would be to draft international standards and /or guidelines on PE that could be adopted by international bodies es. OIE, FAO etc. This would be followed by an integration of PE in the current surveillance system of each country

7.

In terms of this week's questions, again I refer back to other disciplines and how they have managed mixed methods research. We are really talking about combining quantitative information like laboratory test results with qualitative information like community knowledge of disease occurrences. Epidemiology has moved away from tracking qualitative information. Thus surveillance systems only keep record of test results instead of keeping record of the qualitative or meta-information that goes along with that test result. Bringing back the context in which the test was requested, refining the incidence rate of a disease by assessing the impact that the disease had on the community, etc. In the end, participatory epidemiology is not devoid of using quantitative or semi-quantitative data or techniques for getting that data, but rather it is imperative not to sacrifice the qualitative information that the community provides. Triangulation would be a way to see if the 2 streams of epidemiologic information agree or diverge. Thematic analysis of the community knowledge and whether that corresponds with the emphasis derived from traditional laboratory testing would be another.

8.

If I take the case of my country Benin, during avian flu crisis in 2008, and with funding from ILRI and others, under the coordination of Vet Services, we've conducted PDS activities. Thereafter in 2010, as PDS trainer, I had train some public vet service agents. The authorities assured us that, this is a new tool which will therefore be used for collection of animal health data transmitted to OIE (World Organization for Animal Health). But since then to date nothing has changed. Everything agents have learned is set aside. When taking into account this reality, I think in my opinion that the best way should be to go through OIE since it is the institution that manages animal health worldwide. We need a project in partnership with OIE that will be used to train staff of vet services on the importance of PE tools and especially the processing, interpretation and use of collected data.

9.

For us to effectively incorporate PE activities into the official surveillance system I propose that we do a thorough SWOT analysis of the current systems; likewise we do a SWOT of PE then see how best to fuse PE into surveillance systems. Based on personal experience rigidity of most of our surveillance system is their biggest weakness yet the opposite is true with PE which is very flexible in its approach hence can pick even new conditions in the field with minimal resources. It is for this reason I think auditing and reforming surveillance systems to accommodate this PE approaches, quantitative data handling and analyzing will be paramount. For this to be achieved, we need to build bigger teams of PE practitioners, retrain the PE practitioners (refresher courses), Curriculum inclusion of PE.

10.

I think PE activities should be part of active surveillance where a checklist can be devised to assist in understanding of dynamics of diseases, host susceptibility and control options

11.

Here in China, we need to convince the authorities why PE approach is needed by our current surveillance system. Then they need to add PE into annual surveillance program.

12.

The PE activities can well be incorporated within official surveillance systems through raising actor's awareness on the important of PE and in particular the documented amazing results/findings that PE has so far revealed. Actors in this regard are targeting the veterinary investigation centers, universities, policy makers, private practitioners and international development agencies. Investigation centers are doing surveillances and also receiving challenging queries very often and hopeful are well equipped. Universities are doing quite a lot of researches and are delivering batch of graduates yearly. Policy makers are always close to the communities and hence possibility to hear from them, air out for them as well support for the making policy which favors PE recognition in the surveillance systems. Private practitioners are very close to the producers as themselves and as community development projects representatives. International development agencies for livestock sector development including awareness of the diseases identification modalities and control plans which will ultimately favor international livestock market component.

13.

PE could be institutionalized in the same way as the formal/traditional animal health activities/surveillance works. For instance, In Ethiopian case, training the district/sub-district level veterinary offices in this method (PE) (and including pE record sheet in the report) and the trainees train the local leaders/representatives that will help again to inform the public to report any suspect case, any trend/change/deviation from the normal that might indicate the upcoming cases…, this could be an entry point for syndromic (active) surveillance using participatory approach.

14.

This is a rather critical issue. I think we should attempt to provide/show policy makers relevant cases studies where PE has represented an important tool in the hands of local authorities (rather than in the hands of researchers). To my view PE should represent not an alternative to classical surveillance approach (active and passive etc..) but an additional choice in the hands of authorities to be integrated in the routine surveillance efforts when the situation calls for its use. Maybe it has already been organized in the past, but to held a workshop or a scientific forum (i.e. co-organized by FAO/ILRI) where communities representatives, vet authorities and policy makes are represented could be a good starting point. In this occasion, a comparison between communities priorities and animal health policy could be discussed and discrepancies shown. The reasons why discrepancies do exist should be analyzed in detail.

15.

For the PE technique to complement traditional methods of disease surveillance it is necessary to develop projects which will require use of PE technique in disease surveillance for the

countries, international organizations and training institutions. Those project should also have elements of capacity building on which masters and PHD student involved be mandated to use PE techniques into their research. In that way the technique will start to infiltrate into the training institution which will be supervising those student as well as the epidemiology units of those country which will be implementing those projects. For countries and international organization to implement use of PE effectively there is need to institutionalize this technique into countries epidemiology unity by making sure there are legal framework by making sure that they are inconsistency with other existing traditional surveillance techniques. There is an institutional frame work which will take care of budget, office and personnel caring PE on the epidemiology unit and finally there must be administrative frame work which will help on incorporating PE into the existing policy on how PE issues will be addressed on the the policy.

16.

Countries should have a surveillance strategy with clear epi. Office (clearly mention in the organigram of Vet. services) at each levels (central to field).

17.

First and foremost I think the set objective or goal of any surveillance system determines if and how PE will be incorporated in the system whether active or passive. In practice, I always combine the collection of samples for laboratory analyses during PE exercise. My collection of samples are driven by two intent; the first one is based on my objective for the PE and the second is based on the findings provided or implied thoughts by the respondents during the PE exercise. From experience I see laboratory diagnosis as a triangulation tool to further corroborate any existing knowledge or thoughts of the local communities/respondents about disease or any eco-health problem in the area. Samples can include sand, insects (dead and alive), faecal samples, skin scrapings, urine and blood.

18.

The unique and sustainable aspect of the adoption of PE in Nigeria is that the formal non-academic PE training was introduced to government veterinarians as part of the Early Detection Reporting Surveillance: Avian Influenza in Africa (EDRSAIA) the International Livestock Research Institute (ILRI) engaged in a capacity building exercise on Participatory Epidemiology (PE) and Participatory Disease Surveillance (PDS) for Highly Pathogenic Avian Influenza (HPAI) for veterinary personnel in several countries including Nigeria. Thus, as a result of the 2008-2009 Early Detection, Reporting and Surveillance for Avian Influenza in Africa (EDRSAIA) Programme in Nigeria, PDS has officially been integrated into the existing National Animal Diseases Information and Surveillance (NADIS) network and the government veterinarians have diligently adopted, practiced and promoted PE ever since. (Prior to EDRAISA, postgraduate PE training in Nigeria was limited to the Department of Veterinary Public Health and Preventive Medicine, University of Ibadan, Ibadan).

19.

Yes PE activities can augment passive surveillance. They can also be useful during active surveillance especially during disease/case searches or contact tracing following an outbreak. In such instances, lab diagnosis can serve to triangulate the PE findings. For routine

surveillance work, rumor registers can be taken to be part of the PE approach to routine surveillance, provided they are further investigated conventionally.

20.

The institutionalization of PE activities within official system depends upon the broader involvement and ownership by the decision-makers and planners and their exposure to how participatory approaches and methods add value to the existing passive or active surveillance approaches. The flow of policy in public sector is based on "top down" working. Therefore within public veterinary services and veterinary educational institutes the introduction of PE would not be possible unless decision makers/senior faculty had some understanding of PE approaches and methods. It is important that top decision makers may be exposed to understand the value of PE in generating reliable and comprehensive information on disease events and how participatory approaches can help them to formulate pragmatic disease control strategies. Simultaneously mid-level hierarchy may also be taken on board. Field veterinarians may be trained for participatory approaches:

- A series of workshops in different countries may be organized/sponsored by donor agencies to expose decision makers and mid-level managers to PE
- A series of training workshops be conducted at Provincial level within country for field staff to introduce PE
- Findings of PE may be incorporated in routine disease reporting system
- Workshops may be conducted with farmers to provide them information on surveillance system and get the realize taht their participation is important.

The PE findings can be combined with serology or disease reports and based on the information from these approaches the village status "positive' and "negative" can be determined for prevalence of any disease and information derived from both the approaches i-e PE and serology can be compared using 2x2 table method. This will allow to develop pragmatic disease control strategy.

21.

A useful way of integration is to design the PE plan and checklist to match with the objectives of the official surveillance systems, rather than conduction pilot or small scale studies only, however; the method of data collection is different, but at the end the analyzed PE data will convey in the national surveillance information pool, and will be used in supporting the decision making process.

22.

I developed a community based syndromic surveillance system for silvopastoral dual purpose cattle farmers in Mexico. It was a small hypothesis testing Ph.D. project.. It combines some flexibility from PE approaches while describing signs observed, compiling different names for the same sign and developing together the booklet to keep records with the standardization of syndromes and the use of booklets for counts. Each farmer had their own booklet to register every day the number of animals affected by a particular syndrome. Collection and further analysis of booklets provides baseline for syndromes and can be

incorporated into a more extensive surveillance system. This incorporation is what I'm working with right now and I wish you can share different answers from our colleagues Veterinary services were requested after a certain threshold is reached (changes with every disease) thus, collecting samples for an specific diagnosis is under veterinary decision.

23.
PE approaches and activities combine very well with syndromic surveillance activities. The key is facilitating opportunities and mechanisms for exchange between farmers and those responsible for surveillance. New mobile technologies open up many opportunities for this interaction.

24.
PE should be incorporated as a part or a method of surveillance in the official system only when there are enough trained PE practitioners in every veterinary sector.
PE can be incorporated into veterinary epidemiology and veterinary public health training apart from the unique roles of and FAO in refreshing already trained practitioners
I was wondering whether we should not also include the research community in the discussion. For examplewhen you talk about; -Create awareness on the merits of PE among competent authorities (e.g. vet services); Does it include the research community?

25.
Often authorities turn towards the research community to advise them e.g. on the use and design of the 'right' disease surveillance systems, prioritizing diseases, etc. However, participatory approaches and in general qualitative approaches are not always regarded in the research community as being as much valid and strong as pure quantitative approaches. I could imagine that it might hamper the use of participatory in official surveillance systems if researchers don't promote them. This might also influence on the training opportunities in PE. Researchers could provide also valuable input on the limits of PE approaches and its strengths.

26.
PE can be institutionalised through curriculum reviews and training PE to undergraduates students at the University. There is also need to undertake refresher training to extension workers in the country to ensure PE is used while undertaking surveillance activities. It is also important to draft a policy to support the institutionalisation of PE within the delivery of services. In Uganda we have formed an Association of PE practitioners called PARTICPATORY EPIDEMIOLOGY NETWORK IN UGANDA (PENU) to promote PE in surveillance, graduate training and research.

27.
We in Pakistan used PE to confirm absence of Rinderpest during eradication phase. More than 10,000 villages were searched.

28.

Yes. PE was incorporated into the official surveillance system for the control of avian influenza in Nigeria. Although the surveillance was not designed to be so from the outset but eventually PE was included. It is my believe that the inclusion of PE is largely responsible for the success of the AI control/prevention in Nigeria. Inclusion of PE encouraged the sustainability of the system as the people had the sense of ownership of the surveillance system for the public good.

29.
We have done some research on incorporating PE in surveillance. A large study in Egypt found that PDS had quite good sensitivity but low specificity in detection of HPAI. A study of government officers in several developing countries found that some had training and good experience/ perception of PDS but epidemiological capacity was often weak because of insufficient training, support and funding. A study of PDS in Kenya found that PDS was effective and less expensive than conventional epidemiological outbreak investigation. However, it was still not feasible for the government officers to investigate all outbreaks because even the cost of PDS was higher than budgets.

30.
During the implementation of Project in Pakistan by FAO (GCP/PAK/008/EC) 'Support for Emergency Prevention and Control of Main Trans-boundary Animal Diseases in Pakistan" a participatory approach 'PDS" to disease surveillance was introduced and findings were integrated with the findings of Serology and this generated useful information. Unfortunately the use of participatory approaches was not continued after the project.

31.
Thus, as a result of the 2008-2009 Early Detection, Reporting and Surveillance for Avian Influenza in Africa (EDRSAIA) Programme in Nigeria, PDS(/PE) has officially been integrated into the existing National Animal Diseases Information and Surveillance (NADIS) network and the government veterinarians have diligently adopted, practiced and promoted PE ever sinc

32.
I think countries in East Africa notably Kenya and South Sudan have successfully included PE/PDS into their surveillance system. I attach some information for you from a recent presentation that I gave as part of a PE/PDS training for USDA in Southern Africa. In Kenya it did/does work but it has taken time. However as there was a clear need - ensuring freedom of RP. It was actually when conducting PDS for Rinderpest that they found out that they had PPR in Kenya so it definitely did work. Also in South Sudan where government surveillance are still weak they have a pool of people experienced in PE/PDS and they employ it regularly.

33.
In Egypt, the PE program; known as community-based animal health and outreach (CAHO) program; is incorporated in the official surveillance system serving as catalyst for passive surveillance by increasing disease reporting at village level and also serve as an outbreak

investigation tool. The program was initially funded by FAO from 2008-2010 (induction phase), and then handed over to the national veterinary services, and is now totally funded by the government. It is important to keep the number of the PE practitioners reasonable to the financial capacity of the veterinary services.

34. We may need to also engage WHO and not only FAO or OIE.

35.

Yes, OIE and FAO should take more active role in ensuring the use of PE by veterinary authorities. How? Both NGOs should start by using their goodwill to bring the benefit of PE to the vet authorities. They should execute or sponsor projects/studies that is based on PE or the inclusion of PE. They can also organize or sponsor conferences/workshops on use and benefits of PE.

36.

Yes, I do. I think they are best placed to push forward the use of PE. I would also add ILRI to this list of international organizations. They could organize an international workshop where vet professionals, authorities and policy makers interact on PE themes. In addition to this, FAO is particularly best placed as FAO has often direct contact (if not an office located in the same building) with Ministries and Policy makers. They might have an influential role within MoH or MoA (depending on the country).

37.

Sure, these organizations have to play a pushing role as I have suggested in response to basic question number 1.

38.

OIE has already taken a step in the sense that they do recognize PE as a methodology to prove freedom of disease. I think that in combination with laboratory diagnosis PE/PDS is a very valid methodology. I think that it is often a mental thing for many professionals at governments and organizations that PE/PDS is only for developing countries with weak surveillance. This is certainly not the case, just to give you an example. I'm currently advising colleagues in the UK on how to improve their sheep disease surveillance using participatory methods. Often sheep farmers don't keep registers but do have a lot of understanding of disease occurrence, frequencies etc.

39.

I believe with conviction that the OIE has a major role to play in popularizing PE tools. Indeed zoo-sanitary data are collected by the vet services according to the OIE guidelines. It will then be necessary to bring OIE to appropriate PE tools. Then OIE will define with member states a harmonization of points of view with regard to the use of PE tools. Finally, OIE and FAO can then launch a training program for vet services workforce.

40.

Yes, OIE by making it part of the PVS package; FAO by hyping PE role in the eradication of Rinderpest and promoting its application to proposed eradication of CBPP and PPR.

41.
OIE and FAO can publish reference documents about the uses of PE in disease surveillance, outbreak investigation, epidemiological studies, impact assessment, etc.

Comments related with How to deal with possible conflicts of interest between community priorities and animal health policy, e.g. diseases important for the community that are not prioritized by the vet services?

1.
I will answer this under the platform of "Public Health" that includes both human and animal health. We need to leave room for communities to identify their priorities vis-a-vis, the international and national/program priorities. PE tools are so handy when it comes to this setting of priorities. At least I have experience in this.

2.
Proper planning and clear understanding given to the community can minimize conflict of interest.

3.
PE itself can be used to find out the priorities of the communities and perhaps, although a longer process this may be done before it is applied. That way we can tackle both the community priority needs and other national needs for a more effective implementation.

4.
Different stakeholders have different priorities in disease control and also play different, but complementary roles. Thus when priorities differ between communities (private?) and the national veterinary services, control actions should be based on specific stakeholder inputs, with VS priorities being based on “public goods type” of investments while what the community has prioritized could be handled as private good actions by producers and other interest groups.

5.
Each country would be able and free to include additional diseases in his control/eradication /surveillance programmes following the result of PE activities.

6.
Formal epidemiology teaching has focused on structured interviews to fill in boxes or categories rather than accepting and retaining original comments of the interviewee or even reflections of the interviewer. Participatory epidemiology techniques involve re-teaching epidemiologists how to interview and ask questions where you will not get a yes or no, fill in the box, or even simple one sentence answers. PE puts the emphasis on the importance of

information collected back in the hands of the community. However, community-based research runs a fine line between competing interests of community, funding organization and governing bodies. The few projects that I have been involved with, it has been a collaborative process where the community interests are met while allowing for additional interests to be assessed. If this collaboration can be brokered, then the project goes forward. If it cannot, then another funding agency or arrangement has to be made.

7.

It is difficult to manage this conflict. Indeed, what is a priority for a community in one region of the country may not be so for another community in another region of the same country. However, vet services apply a national animal health policy. Therefore, a better approach to this conflict management will be to make a country's zoning and identify priorities in each area. In other words, the authorities in charge of vet services, should establish a decentralized animal health policy.

8.

The lack of knowing priority areas for farmers, I believe emanates from the systemic failures in extension services which can be associated with myriad of factors e.g lack of support for extension workers by government, low staffing level. If extension workers (read animal health practitioner) aren't close to farmers they will hardly know what really affects the farmer hence to top bottom approach in prioritizing disease. For us to move out of this, I think we need to be better mobilizes of resources that can support closer farmer to veterinarian interactions possibly through lobbying government or partners in this venture. We can also take advantage of various other government programs like human polio campaign that are conducted on a door to door approach, vets can join this field teams to interact and interrogate the communities in order to appreciate their concerns in framing animal health policy, intervention and control programs.

9.

PE will ensure that interests of communities are understood and VS could see how best harmonization can be done such that priorities take into account interest of communities to increase compliance

10.

My view: In my opinion, it is the government's duty to list priorities disease. Especially those disease related to public health but not worth to control in agricultural animals, like H7N9 avian flu, Hepatitis E in pig farms.

11.

Could be a golden opportunity to compromise the gap. Previously, priorities of the community are less known. If we could incorporate the interest of the community to the

background/motives of priorities in the animal health policy, then the implementation would be more effective. That could be one of the reasons why most animal health policy implementation without the community interest wasn't successful as such. So, to me there would not be conflict rather synergy to each other.

12.

Again, the objective of the PE should set out clearly the disease(s) to work on. Importantly, working on disease of high priority to the targeted community will support sustainability of the surveillance system and/or the control options. As such it is important to have a clear insight into the major problem(s) of the community; a participatory rural appraisal exercise can achieve this preliminary background check. Now, with this information, there are two (2) possible ways to prevent and/or manage any conflict of interest between community priorities and animal health policy. 1- Here the animal health policy is explained clearly to the relevant communities as the targeted objective. Then as incentive, one or more of the community high-priority health problems will equally be addressed alongside the execution of the animal health policy. I believe this is the best way to deal with the conflict; that is, if any between the community and animal health policy as every involving stakeholders will share in the ownership of the system. 2- Second option is to combine one or more priority problems of the targeted respondents together with the animal health policy such that they are inseparable from each other, that is, it will be seen together as the animal health policy. The challenge here is that this combination can only exist when it is pertinent to combine the two conflicting priorities as one. In this case there is no need to define to the relevant communities the differences between community priority problems and the animal health policy.

13.

Animal Health policies are supposed to be determined by and reflect community priorities. Such Animal health policies must then accommodate community livelihood priorities, not just economic or international trade considerations.

14.

Ideally there should be no conflict between community prioritization and policy. This is because policy should be stakeholder based. However, this takes place in practice because the community looks at the immediate need or perceived immediate losses while the policy implementors have a wider and more long term view. Stakeholder educations and participation of communities towards policy implementation can bridge this gap. This is where PE comes in.

15.

It happens rarely but again this can be solved through conducting workshops with farmers to provide them information on surveillance system and get the realize taht their participation is important and develop good linkages with the communities.

16.

In my opinion, most important diseases for the community ARE the most important objective of our work. With community engagement, we can then incorporate other diseases of public health importance or trade importance.

17.

At the end of the day, if farmers are to have confidence in the veterinary services need to be responsive to community priorities. The response may vary from simple acknowledgement of community priorities all the way through to active responses on the ground.

The HPAI H5N1 pandemic provides a good example of the importance of understanding community priorities as a sole focus on HPAI has not resulted in a sensitive surveillance system. For HPAI control to be effective and efficient, it needs to be done in the context of all poultry diseases that are differential diagnoses for HPAI. Further information on this is available in:

- Alders, R.G. and Bagnol, B. 2007. Effective communication: the key to efficient HPAI prevention and control. World's Poultry Science Journal 63:139-147
- Gardner, E. and Alders, R.G. 2014. Livestock Risks and Opportunities: Newcastle Disease and Avian Influenza in Africa. Planet@Risk (Special Issue on One Health) 2(4):208-211

18.

Call upon the forum for the two social gatherings and apply some of the PE tools. I hope will decide for them as PE goes with reasons from the grass root.

19.

Naturally, the priorities of the community and the authorities must be different because the overall objective of both are different. But it can be aligned and both parties can meet at a common point. One of the unique roles of PE is that it makes veterinary services available to the communities. Places where we conducted disease surveillance, veterinary services were made available to them because they became more aware of their challenges and were ready for an intervention. So using PE as an active surveillance will make veterinarians more active in the rural communities.

21.

What about conflict between interest of the International community and animal health policy in country?

22.

I agree with parts of both responses. It is natural and not negative whenever official and community priorities are different but I don't agree that this means there is a disconnection between the two. This happens every time even among friends. Where there will be problem or failure is if the National or official system fails to incorporate some or all community priorities into the list of official priorities, while importantly ensuring that different approaches are utilized in different parts of the country. I agree to the fact that this merging of priorities will ensure the sustainability of the system.

23.
I support the second one.

24.
I am in support of view No. 2.

25.
View No. 2.

26.
More inclined towards the second. This doesn't mean that the government should forget about their priority diseases because of e.g. trade implications but information from PE could inform the strategies for overall government surveillance and animal production activities. Often tickborne diseases such as heartwater come up first in the most important diseases, if this comes up consistently in a number of areas the government should consider doing something about it. the vaccine is still complicated but at least more efforts should be put on awareness of tickborne diseases and dipping in general. This doesn't imply that the government should provide free dipping as was often done in the past (and still in some countries) but livestock keepers should be made aware. often people really don't know that the disease is transmitted by ticks.

27.
Those who think that the non-matching between community and official priorities is due to a disconnection between the parties involved, i.e. the national authorities don't understand the reality of the communities. These contributors advocated for incorporating some of the community priorities (found through PE) into the list of official priorities, while also ensuring different approaches are utilised in different parts of the country. By doing so, national programs will be more successful because they will be targeting the real needs of regional communities.
I think it is better to promote PE in developed countries , a sure way to get attention from the developing countries as well. I also agree the last point "-PE can effectively contribute to a better management of animal health by breaking the gap between communities and authorities". In addition I would like to point out that there should be a n attitude change in officials when it comes to practicing PE, which I believe can be achieved by proper PE training.

28.
We may start to partner with main stream epidemiologists like in established platforms like WHO or FETPs to build a bigger PE workforce. We may use the same platforms and scientific forums to showcase the applications of PE in mainstream surveillance.

29.

Capacity building of the ground staff to conduct PE will remain very crucial. How it can be done in a country may differ from region to region and country to country depending on the populace demography.

30.

Equipping mainstream vet staff with PE can significantly improve both their understanding of stakeholder perceptions on disease control and communication on government policy regarding the control of public good diseases.

31.

It would be interesting to discuss the use of PE methods also in developed countries.

32.

Nowadays with One health concept, PE tools need to be disseminated in the public health workforce. This will allow its large use.

33.

I think we need first to consider PE approachs, to correlated animal disease and stackeholders income . Disease could be important in community because in case of outbreak, people could not go easily to market, so animals are died (lose money)
Second correlated animal disease and zoonotics aspects (de ath rate, disability to work ...
So we need to prioritized disease according to income, incidence, disability... and find appropriate way to investigate, to riposte, to control.
So what useful approach we need to implement to provide data or to analyse data, behaviours, etc. For example in ebola response in Dubreka where I was deploied for 2months , in community where reluctance is recurent, Its possible to implement. PE approach to investgate cases , contact tracing and highlight why people refuse are unwilling to accept scientist approach, swab for good diagnostic to control disease. Classical epi could noticed this fact but did not explain. In conclusion according to the diseases clunicaly, to the step of investigations, économic importance we need classical, participatory and other field tools to control diseases.

34.

Need to develop guidelines such that disease search reports are comparable.

35.

PE is an unreplaceable method in epidemiological research. However, many experts especially in China, they misunderstand what PE can be used for, many of them mistakenly assume that PE is only suitable to less developed countries where Animal husbandry and veterinary system is not well established. So at least in China, the highest priority to introduce PE to different stakeholders.

36.

Sharing at the end the proceedings of this forum.

37.

Incorporating community involvement/priorities/opinions to the formal veterinary service in terms of diagnosis, surveillance, ...and in general to the animal health policy contribute positively to its effectiveness in terms of implementation and so to the final outcome.

38.

CAHWs are a part of the network or not? If yes, to which level we can rely on a farmer (CAHW) to report disease in his/her village with a possible (negative) consequences for the community?

39.

Participation of livestock farm owners and rearers in disease identification, reporting, monitoring, surveillance, prevention and control, whether on small backyard scale or on large scale commercial, is the basic principle of PE.

40.

The role of PE should be emphasized in veterinary schools . Not only in third world countries but also in the rest of the world.

41.

It is important that the use of PE may be promoted as suggested in my response to basic question No. 1, people from human health sector, planning and monitoring sector (as PE approaches can also be used for impact assessment of the projects). The capacity building to practice PE of public veterinary services is of prime importance, this will generate the specialized experts with good PE skills. The promotion of PE with concerned in developed world will open new venues for the use of PE approaches. PE is most pragmatic approach to break the ice between communities and veterinary services.

42.

A BUILD UP ON THE PE EFFORTS OF ILRI PARTICIPATORY EPIDEMIOLOGY OF NETWORK OF ANIMAL AND HUMAN HEALTH (PENAPH) 2. A STUDY ON HOW PE GOT OFFICIALLY INTEGRATED AND ACCEPTED INTO NIGERIA'S GOVERNMENT VETERINARY SERVICE as a result of the 2008-2009 Early Detection, Reporting and Surveillance for Avian Influenza in Africa (EDRSAIA) Programme in Nigeria 3. A WORLD-WIDE LIST OF PE PRACTIONERS (A.K.A PENAPH LIST)

43.

PE is a good tool but the challenge has been in managing data. Usually PE studies generate a lot of data during research studies. Analysing such data is very difficult which leads to loss of interest in using PE. There is need to train users in managing and analysing PE data.

44.

Perhaps to make the participatory diagnosis in animal diseases and having obtained the perceptions of the presence of diseases in the community, this information could be compared to the information held by health authorities regarding the clinical and laboratory diagnosis of these diseases in their community or nearby communities. With this exercise perhaps might

say that diseases prioritized in the community are very likely to be those who have been diagnosed clinically or laboratory in close communities. With this information that authorities must include in their control plans or policies the control of these diseases. This situation usually happens in countries where the official veterinary service cannot reach all the communities and their surveillance systems are lack off. Therefore, the reporting of diseases or their presence in these communities is underestimated and wrongly delimits the area presence of these diseases and the strategies could not reach these communities. PE would be a great tool, as this will allow forgotten communities for the veterinary services to express their needs, context and As a result they will be visible. This could be an interesting way to enable the authorities to listen they needs and how on the basis of their culture or nanomedicine this communities could be solved their needs. Furthermore, this dialogue would perhaps improve national strategies for disease control based on the management of their herds in the community. So, these control plans might be adapted according of the operating systems how animals in their communities rise. These adapted strategies can be applied without having a conflict between national authorities and communities. This partnership would allow communities to be trained on the control of these diseases with the objectives of the veterinary service. Finally it could be improve the surveillance system because the communities which not only communicate the presence of the priority diseases, but the presence of other emerging or reemerging diseases, that may have capacity to generate epidemics in animal health and veterinary public health.

45.

One of the example is in pastoral communities from which, government and in collaboration with development organizations(donors) concentrate on controlling the tick borne diseases while forgetting the production and consumption practices which expose respective communities into great chances of contracting zoonotics among others; anthrax, and TB. Though involvement of producers (community members) from which, PE application will allow detail identification and prioritization of the challenges facing them and their control strategies including possible zoonotics in the production consumption chain

46.

Federal Ministry of Agriculture and Rural Development, Abuja, Nigeria. Through participatory disease surveillance, we were able to establish the major livestock species kept and disease of priority in different communities in Plateau State, Nigeria. Most of the communities had poultry ranking first with regards to the livestock species mostly kept and the disease of utmost importance. However in the course of the exercise, one particular community differed in the livestock species kept and of course the disease of utmost importance. This tells us that a community is likely to have a different need with regards to disease control than what is assumed their need it. PE is therefore a needful tool to help government and the farmers to control the disease that is of importance to the different farming communities. The only challenge will be that different diseases have to be in the control programme. I think that farmers/communities could assure that their needs are being taken into consideration and addressed in disease control strategies and livestock policies by forming a cooperative and

making their views known to Government and at the same time contributing their own quota in animal disease control rather than leaving it in the hands of Government alone.

47.

From your experience can you give examples of such diverging disease priorities? How do you think farmers/communities could assure that their needs are being taken into consideration and addressed in disease control strategies and livestock policies? In my experiences in Kenya and Somalia, the following somehow "positive" example shows the findings of PE exercise implemented under an ECHO emergency project aiming to assess livestock health in one specific district (Garissa) in Kenya. The PE survey teams were always formed by veterinarians from the vet services and external experts. We reproduced this exercise in different districts in north eastern Kenya. Following this exercise, in partnership with the Kenyan veterinary services, we integrated this information with previous evidence of diseases presence in the district, and we purchased vaccines (according to the different PE ranks). We then implemented the vaccination campaigns which targeted different diseases in different districts. However, the project (and the Veterinary Services), for a number of reasons (mostly economic and practicability of actions) haven't implemented any actions towards the control of the tick borne diseases or trypanosomiasis which ranked very high in all districts for a number of species. This was the limitation of this exercise. If such an approach is regularly used by vet services and the findings of PE are considered reliable enough by policy makers (but we would need to define "enough" here), PE could be the basis for targeted strategies applied to animal health control. What is needed is the evaluation of the effectiveness of control measures based on PE results.

CATTLE	GOAT	SHEEP	DONKEY	CHICKEN
**1) Tryps.**<br>**2) TBD**<br>3) CBPP<br>4) FMD<br>5) LSD<br>6) BQ<br>7) Worms<br><br>**CBPP**:<br>Kotile,<br>Corissa,<br>Hara,<br>Jalish,<br>Handaro	**1) CCPP**<br>**2) TBD**<br>3) Tryps.<br>4) Mange<br>5) Worms<br><br>**TBD:**<br>Major problem in:<br>Gababa,<br>Kotile,<br>Jalish	**1) Enterotox.**<br>**2) TBD**<br>3) Tryps.<br>4) Worms	**1) Tryps.**<br>2) Worms	**1)Ectop**<br>(lice, mites)<br>2) Coccidiosis

48.

While PE is very vital in rare and exotic disease surveillance, endemic and production disease stand a very little position however, can help to build an understanding the immediate and long impact of the disease. Our experience on farmer driven study request on blindness on cattle through a condition - IBK revealed a very astonishing results based on PE tools. Although

its clear the causative agent in Bacterial Morexella spp, the vector agent responsible was very surprising. A nocturnal insects which has been rarely reported in Africa(Uganda and Ivory Coast)proved to be main the culprit. The insects are tiny and rarely seen during the day - they only land on the animal eye during the night and later disappear. You need to spend a night at cattle boma to be able to see them. It was very appreciated study output considering that farmer new very little about the fly role. Such information was an eye opener to the veterinary authorities both at local scale, regional and national scale on how to address and set priorities

49.

An example of diverging priorities between smallholders and governments would be FMD, Farmers/communities should associate (and be supported to do it) and lobby national governaments. PE needs to be integrated as tool to deal with animal and human diseases. One possible approach would be first to set international standards and guidlines, then set up a framework policy and last to implement it at national level

Topic 3

Where should priorities lie for developing PE in the future?

1.

- Explaining need for blinding
- Ensuring satisfaction of the community and the field
- Reducing bias in subject recruitment

2.

Whereas PE has been acknowledged as a necessary tool for epidemiology, a lot of times PE is used in a very limited way depending on how and why practitioners were trained. In some instances practitioners are not able to differentiate between PE and PDS. My understanding is that PE is the overarching participatory approaches and methods/tools under which various other focused tools such as PDS, participatory baseline and impact assessments of livestock projects, participatory risk assessments, participatory disease socio-economic studies and general disease studies are delineated from. The PE focus on rural illiterate pastoral communities does not hold any longer (Harding et al, 2014). Therefore one priority in PE future development is to gather and analyze evidence of various uses of PE globally. This information should be used to update the existing PE, PDS and Participatory impact assessment manuals with a view of creating one all inclusive PE training manual that is composed of a basic introductory training module and other specialized training modules.

3.

- Validation of the methods by comparing to other conventional epidemiology methods and lab procedures.
- Developing stronger statistical tools.

- Journal/society of PE.

4.

On the point of sustainability of PE which was alluded to, my contribution is that PE should be mainstreamed strongly in curricula of veterinary schools. Most veterinary schools do not have PE component strong in the Epidemiology modules.

5.

- Lobbying on OIE and FAO top authorities.
- Lobbying on vet services top authorities from OIE member States.
- Training of vet service agents.

6.

Haven concluded on a holistic definition and how to foster PE in the global system, the objective of future developments of PE becomes clear and focused. This makes the task easy.
PE is not an emerging field, as we all agreed. This means it has always been in use either unknowingly or knowingly. I believe the former is more common than the latter, hence the general thinking that this is an emerging field. The issue at stake here is sustainability of PE-driven projects, whether it is disease control and prevention or maintenance of public health/eco-health. Since PE is not an emerging field, as part of future developments of PE, all we need do is redirect the public's (or all relevant stakeholders') attention and focus to PE and its benefit. Particularly as we have glowing examples of the successful application PE to work with. This leaves out sustainability as main priority for future developments of PE. Sustainability: this is only possible when all relevant and actively participating stakeholders are given the ownership opportunity of the PE-driven project. In effect, the major focus of development of PE is in deciding the objectives/benefits (that will ensure its sustainability) as regards its application in projects. To this end, I think future developments of PE should be focused on the developments and standardization of step-wise checklist that can guide the formation of objectives for the application of PE in any project in question. Importantly, since, science, people, environment and ecology, etc, are ever dynamic and sometimes unpredictable; the final approved checklist should be reviewed at regular interval or as deemed necessary. The regular review of the checklist will ensure its relevance over time or space. NB: This checklist is to guide towards forming objectives that will ensure the sustainability of any project that is PE-driven, bearing in mind that sustainability will be assured when all stakeholders are included in the consideration process. Based on my humble experience, I think the checklist should include:

- Define or identify the targeted stakeholders. This can be the host community or subset of a population/continent, professional bodies or association and/or even the government(s).
- Define or identify other stakeholders that are anticipated to be participating in the project (that is those not captured in 1 above).
- Is government(s) involved or not?
- If government(s) is/are involved, any conflict of interest?
- Who is/are the sponsors of the project?

- Does the sponsor have any conflict of interest?

7.

- Write up examples of institutionalisation of PE.
- Provide information on feasibility, accuracy and cost:benefit.
- Strengthen communities of practice.

8.

Priorities for PE development:

- Review PE training curriculum and materials
- Conduct refresher trainings
- Train more PE practitioners
- Support integration of PE in routine surveillance
